# Translational GTPase BipA Is Involved in the Maturation of a Large Subunit of Bacterial Ribosome at Suboptimal Temperature

**DOI:** 10.3389/fmicb.2021.686049

**Published:** 2021-07-13

**Authors:** Kwok Jian Goh, Rya Ero, Xin-Fu Yan, Jung-Eun Park, Binu Kundukad, Jun Zheng, Siu Kwan Sze, Yong-Gui Gao

**Affiliations:** ^1^School of Biological Sciences, Nanyang Technological University, Singapore, Singapore; ^2^Singapore Centre for Environmental Life Sciences Engineering, Nanyang Technological University, Singapore, Singapore; ^3^Faculty of Health Sciences, University of Macau, Macau, China

**Keywords:** BipA, ribosome biogenesis, large subunit maturation, conditional protein expression, stress response, suboptimal temperature growth

## Abstract

BPI-inducible protein A (BipA), a highly conserved paralog of the well-known translational GTPases LepA and EF-G, has been implicated in bacterial motility, cold shock, stress response, biofilm formation, and virulence. BipA binds to the aminoacyl-(A) site of the bacterial ribosome and establishes contacts with the functionally important regions of both subunits, implying a specific role relevant to the ribosome, such as functioning in ribosome biogenesis and/or conditional protein translation. When cultured at suboptimal temperatures, the *Escherichia coli bipA* genomic deletion strain (Δ*bipA*) exhibits defects in growth, swimming motility, and ribosome assembly, which can be complemented by a plasmid-borne *bipA* supplementation or suppressed by the genomic *rluC* deletion. Based on the growth curve, soft agar swimming assay, and sucrose gradient sedimentation analysis, mutation of the catalytic residue His78 rendered plasmid-borne *bipA* unable to complement its deletion phenotypes. Interestingly, truncation of the C-terminal loop of BipA exacerbates the aforementioned phenotypes, demonstrating the involvement of BipA in ribosome assembly or its function. Furthermore, tandem mass tag-mass spectrometry analysis of the Δ*bipA* strain proteome revealed upregulations of a number of proteins (e.g., DeaD, RNase R, CspA, RpoS, and ObgE) implicated in ribosome biogenesis and RNA metabolism, and these proteins were restored to wild-type levels by plasmid-borne *bipA* supplementation or the genomic *rluC* deletion, implying BipA involvement in RNA metabolism and ribosome biogenesis. We have also determined that BipA interacts with ribosome 50S precursor (pre-50S), suggesting its role in 50S maturation and ribosome biogenesis. Taken together, BipA demonstrates the characteristics of a *bona fide* 50S assembly factor in ribosome biogenesis.

## Introduction

The ability of bacteria to respond, adapt, and grow at suboptimal temperature is known as cold shock response, which is activated in *Escherichia coli* under a condition of a sudden drop in culturing temperature (usually from 37 to 15°C) (Barria et al., 2013). Suboptimal temperature can cause both physiological and morphological changes in the bacteria and even cell death if the temperature shift is beyond bacterial tolerance. The majority of cold-inducible proteins involves RNA metabolism, indicating that regulation of RNA metabolism is crucial for suboptimal temperature adaptation (Barria et al., 2013). The most notable examples include the following: (i) cold shock protein (Csp) family proteins, which are RNA chaperones and able to prevent formation of RNA secondary structures (Phadtare et al., 2003; Barria et al., 2013; Uppal and Jawali, 2015); (ii) DEAD-box RNA helicase (DeaD) that can unwind RNA secondary structure to promote degradation (Charollais et al., 2004; Prud’homme-Généreux et al., 2004; Resch et al., 2010); and (iii) RNase R, the only 3′–5′ exonuclease in *E. coli* that degrades double-stranded RNA without the help of a helicase (Cairrão et al., 2003; Awano et al., 2010; Barria et al., 2013).

The translational stress response involves a key component of protein-synthesizing machinery, the ribosome. Bacteria utilize a series of protein factors that bind ribosome to modulate translation in order to cope with stress. For example, the RelA/SpoT homolog (RSH) proteins bind ribosome when there is a surge of uncharged tRNAs caused by the shortage of amino acids. Consequently, binding of RSH to ribosome triggers the alarmone synthesis and is followed by the stringent response (Hauryliuk et al., 2015). Notably, there are two proteins that bind to the ribosome to exert their stress response function: ObgE and BPI-inducible protein A (BipA). ObgE is an essential GTPase, and its homologs have been ubiquitously found across all kingdoms of life (Sato et al., 2005; Jiang et al., 2006). An analysis of those immature 50S particles, accumulated in cells with the ObgE depleted, showed that late assembling r-proteins (L33, L34, and L16) were under-represented, indicating that ObgE is an important factor during the late step of 50S biogenesis (Jiang et al., 2006).

BipA has been implicated in various functions of pathogenic bacteria, such as *Salmonella typhimurium* (*S. typhimurium*), *Pseudomonas aeruginosa* (*P. aeruginosa*), and enteropathogenic *E. coli* (EPEC). BipA is upregulated by ∼7-fold when *S. typhimurium* is “attacked” by bactericidal/permeability increasing (BPI) antimicrobial peptide (Qi et al., 1995). BipA is also known as TypA, referring to tyrosine phosphorylated protein A, and this is the case in *P. aeruginosa* where TypA was found to be involved in virulence, antimicrobial resistance, and biofilm formation (Neidig et al., 2013). In EPEC, BipA has been reported to upregulate the virulence and reduce flagella-mediated motility (Grant et al., 2003). The *bipA* deletion would result in growth delay of *E. coli* K12 strain while cultured at low temperature (e.g., 20°C) (Pfennig and Flower, 2001). Furthermore, Choudhury and Flower (2015) reported that the ribosomal particle distribution of the *bipA* deletion strain changes dramatically, with the accumulation of the 30S and the presence of 50S precursor (pre-50S), indicating a possible role of BipA in ribosome biogenesis (Choudhury and Flower, 2015). Interestingly, a transposon-mediated random insertion mutation into *E. coli* K12 strain genome revealed that the disruption of the *rluC* gene can suppress the phenotype of the *bipA* deletion (Krishnan and Flower, 2008). RluC is a pseudouridine synthase, which converts three uridines (U955, U2504, and U2580) in 23S rRNA to pseudouridines (Ψ955, Ψ2504, and Ψ2580). Pseudouridine is a uridine isomer where the uracil base is linked to the pentose sugar by a carbon-to-carbon bond, resulting in the ability to form an additional hydrogen bond and thereby increasing the stability compared to uridine (Kierzek et al., 2013). In addition, Choudhury and Flower (2015) discovered that the deletion of DeaD exacerbates both the growth and ribosomal particle distribution defects (Choudhury and Flower, 2015). Since DeaD is involved in 50S biogenesis, it is not surprising that *bipA* and *deaD* double mutations could cause an evident defect in ribosome biogenesis. Crystal structures of the apo-, as well as the nucleotides [GDP, (p)ppGpp, and GDPCP] bound BipA, showed no significant difference in their overall conformations (Fan et al., 2015; Kumar et al., 2015). However, a drastic conformational change was observed in domains III, V, and the C-terminal domain (CTD), upon GTP form BipA binding to 70S ribosome (Kumar et al., 2015). The conformational change of the CTD was particularly interesting because the CTD loop extends into the ribosomal A site to establish extensive contacts with the tRNA acceptor stem; however, the precise role of such interactions is largely unknown (Kumar et al., 2015). Nevertheless, the structure of BipA with ribosome demonstrated an activated form of BipA in the ribosome, with the catalytic residue, histidine 78 (H78), situated close to the 23S rRNA sarcin–ricin loop (SRL) in a ratcheting ribosome as well as interacting with the bound nucleotide (GDPCP). Collectively, BipA is an authentic ribosome-dependent trGTPase. However, how BipA in association with 70S ribosome is correlated with bacterial stress response as well as how BipA functions in conditional translation for bacterial cells at suboptimal temperature require further studies.

Notably, recent quantitative mass spectrometry data nicely showed the accumulated pre-50S intermediates in *bipA* mutant cells at suboptimal temperature, with several r-proteins absent, further demonstrating the role of BipA in 50S subunit assembly (Gibbs et al., 2020). Furthermore, a paper published during our manuscript preparation revealed that bacteria can remodel their protein expression relevant to biofilm formation in a temperature-dependent manner by modulating BipA abundance (Del Peso Santos et al., 2021). With the desire to support the ongoing effort in BipA-related studies and to expand our efforts on structural study of ribosome-associated proteins and biofilm formation as well as its relevant pathogenesis and resistance (Tanaka et al., 2008; Selmer et al., 2012; Kumar et al., 2015; Yu et al., 2015; Ero et al., 2016; Kumar et al., 2016; Yang et al., 2017), here we report additional evidence to demonstrate the role of BipA in ribosome biogenesis, specifically in large subunit 50S maturation, and conditional protein translation at suboptimal temperature through combinative approaches. In particular, our findings by tandem mass tag (TMT)-based quantitative proteomic analysis identified proteins relevant to RNA metabolism (e.g., DeaD) with increased expression levels upon BipA deletion, such an effect can be suppressed by a further mutation of RluC or complementation by BipA. The upregulation of these protein possibly indicates that bacterial cells can compensate for the loss of BipA during 50S biogenesis.

## Materials and Methods

### Bacterial Strains and Culturing

The *E. coli* strains used in this project are listed in Table 1. Strains were grown in Lysogeny broth (LB) at 37°C for optimal growth and at 25°C for suboptimal growth. The antibiotics chloramphenicol (34 μg/ml), gentamicin (15 μg/ml) and kanamycin (30 μg/ml) were added when required for selection.

**TABLE 1:** Strains of *E. coli* and plasmids used in this project.

*E. coli* strains	Description of the genotype	Reference or sources
K12WT	K12 BW25113 wild-type; Δ*(araD-araB)567*Δ*lacZ4787(:rrnB-3)*λ*- rph-1*Δ*(rhaD-rhaB)568 hsdR514*	Baba et al., 2006
Δ*bipA*	F-, Δ*(araD-araB)567*, Δ*lacZ4787(:rrnB-3)*, λ*-, rph-1*, Δ*bipA733:kan*, Δ*(rhaD-rhaB)568*, and *hsdR514*	Baba et al., 2006
Δ*rluC*	*rluC:: Gen^R^*; gentamicin resistance cassette replaced entire K12WT *rluC* gene using pRed/ET	This study
Δ*rluC/*Δ*bipA*	*rluC:: Gen^R^*; gentamicin resistance cassette replaced entire Δ*bipA rluC* gene using pRed/ET	This study
Δ*relA*	F-, Δ*(araD-araB)567*, Δ*lacZ4787(:rrnB-3)*, λ^–^, Δ*relA782:kan, rph-1*, Δ*(rhaD-rhaB)568, hsdR514*	Baba et al., 2006
Δ*relA*/Δ*spoT*	Homologous recombination gene replacement using upstream and downstream sequences of *spoT* on Δ*relA*	This study
Δ*relA*/Δ*spoT*/Δ*bipA*	Homologous recombination gene replacement using upstream and downstream sequences of *bipA* on Δ*relA* Δ*spoT*	This study
*obgE*:: pVIK111	K12WT; in frame *obgE-lacZ* translational fusion	This study
Δ*bipA obgE*:: pVIK111	Δ*bipA*; in frame *obgE-lacZ* translational fusion	This study
Δ*bipA obgE*:: pVIK111 + *bipA*	Δ*bipA obgE*:: pVIK111 harboring pCA24N-BipA	This study
Δ*rluC/*Δ*bipA obgE*:: pVIK111	Δ*rluC/*Δ*bipA*; in frame *obgE-lacZ* translational fusion	This study
*deaD*:: pVIK111	K12WT; in frame *deaD-lacZ* translational fusion	This study
Δ*bipA deaD*:: pVIK111	Δ*bipA*; in frame *deaD-lacZ* translational fusion	This study
Δ*bipA deaD*:: pVIK111 + *bipA*	Δ*bipA deaD*:: pVIK111 harboring pCA24N-BipA	This study
Δ*rluC*/Δ*bipA deaD*:: pVIK111	Δ*rluC/*Δ*bipA*; in frame *deaD-lacZ* translational fusion	This study
MC1061 (λpir)	*thi thr-1 leu-6 proA2 his-4 argE2 lacY1 galK2 ara-14 xyl-5 supE44 pir*	Zheng et al., 2007
**Plasmids**		
pCA24N-BipA	ASKA clones harboring *bipA*, Cam^R^	KEIO collection
pCA24N	Modified from pCA24N-BipA, harboring no insert to act as an empty plasmid, Cam^R^	This study
pCA24N-BipA_H__7__8A_	Point mutation to substitute H78 with alanine, Cam^R^	This study
pCA24N-BipA_H__7__8Q_	Point mutation to substitute H78 with glutamine, Cam^R^	This study
pCA24N-BipA_T__544_____55__2d__el_	CTD loop in frame truncation of 9 amino acids (amino acids T544 to D522), Cam^R^	This study
pDS132	pCVD442 modified suicide plasmid, *pir* dependent, *sacB*, Cam^R^	Philippe et al., 2004
pDS132-*bipA*	pDS132 carrying homologous arms (−552 to 7; 1,803 to +730) of *bipA, sacB*, and Cam^R^	This study
pDS132-*spoT*	pDS132 carrying homologous arms (−529 to 2; 2,073 to +713) of *spoT, sacB*, and Cam^R^	This study
pVIK111	Contains *lacZ* for translational fusion, Kan^R^	Kalogeraki and Winans, 1997
pVIK111-*obgE*	pVIK111 carrying 3′ region (623–1,172) of *obgE*, in frame with *lacZ*, Kan^R^	This study
pVIK111-*deaD*	pVIK111 carrying 3′ region (1,295–1,889) of *deaD*, in frame with *lacZ*, Kan^R^	This study

Deletion of *rluC* gene was carried out using the pRed/ET system (Heermann et al., 2008), replacing *rluC* with gentamicin resistance cassette through sequence-specific homologous recombination. Linear DNA fragments consisting of the homologous arms (50-bp sequences upstream and downstream of *rluC*), sandwiching the gentamicin antibiotic cassette, were transformed into pRed/ET-harboring *E. coli* K12 strain (K12WT) using electroporation.

Deletion of genomic *bipA* and *spoT* genes was carried out using the pDS132 (Philippe et al., 2004) suicide plasmid. Approximately 500 bp upstream and downstream of *bipA* and *spoT* were amplified as the homologous arms. The upstream and downstream arms were combined through restriction site ligation and cloned into pDS132 to obtain pDS132-*bipA* and pDS132-*spoT* plasmids. The plasmids were transformed into target cells using electroporation and two rounds of selections. Kanamycin-resistant cells were selected from the first round of selection, and sucrose-sensitive cells were selected from the second round of selection.

Knock-in of *lacZ* downstream of *deaD* and *obgE* was done using pVIK111 (Zheng et al., 2007) suicide vector. In-frame insertion was created by amplifying approximately 500 bp of 3′-end of the target gene, while excluding the stop codon, and cloned into pVIK111 yielding pVIK111-*deaD* and pVIK111-*obgE*. The plasmids were then transformed into *E. coli* K12 strain (K12WT), Δ*bipA*, Δ*bipA* (pCA24N-BipA), and Δ*rluC/*Δ*bipA* using electroporation. Kanamycin-resistant clones were selected.

### Site-Directed Mutagenesis

Modifications of pCA24N-BipA were done using the QuikChange method to produce pCA24N-BipA_H__7__8A_, pCA24N-BipA_H__7__8Q_, and pCA24N-BipA_T__544___*D*__55__2d__el_. Primers were designed to have equal lengths of nucleotides extending toward 5′- and 3′-ends from the point of modification. Briefly, the first step was PCR amplification of pCA24N-BipA to produce two single-stranded circular DNAs by carrying out the forward and reverse amplification in separate reactions. Secondly, the samples were mixed in the presence of 1 μl *Dpn*I enzyme (NEB^®^), which digested the template DNA. Next was activation at 37°C for 2.5 h, followed by deactivation at 80°C for 20 min, and then denaturation at 98°C for 15 min. Lastly, samples were incubated at room temperature to allow the denatured products to re-anneal. Chloramphenicol-resistant clones were selected for sequence verification.

### Growth Assay

Growth curves were determined using a 96-well microplate. The overnight cultures were diluted in LB to OD_600_ ≈ 0.02 in a final volume of 100 μl/well. Plates were shaken in a shaker incubator at 200 RPM and temperature of 37°C for optimal growth and 25°C for suboptimal growth. The cell density was measured by a TECAN Spark^TM^ 10M multimode microplate reader without microplate lid every half an hour (37°C) or every hour (25°C) under the settings of 600-nm light absorbance, 25 flashes, and 120-ms wait time between wells. Growth curves were performed in biological triplicates.

### Swimming Motility Assay

Overnight cultures were diluted to OD_600_ ≈ 1.0 in 0.9% (*w*/*v*) NaCl. Soft LB agar (0.3% *w*/*v*) was inoculated with overnight cultures by using a drawing needle to dip into the diluted overnight cultures and then pierced through the soft agar to the middle. The plates were incubated at room temperature for 24 and 48 h. Results were recorded in the form of images and measurements of swimming diameter.

### Sucrose Gradient Sedimentation Analysis

Cell pellets [K12WT, Δ*bipA*, Δ*bipA* (pCA24N-BipA), Δ*bipA* (pCA24N-BipA_*H*__7__8A_), Δ*bipA* (pCA24N-BipA_H__7__8Q_), Δ*bipA* (pCA24N-BipA_T__544___*D*__55__2d__el_), Δ*rluC*, and Δ*rluC*/Δ*bipA*] were resuspended in RNA lysis buffer [20 mM HEPES pH 7.5, 10.5 mM MgOAc, 100 mM NH_4_Cl, 0.5 mM EDTA pH 8.0, and 6 mM β-mercaptoethanol (β-ME)] in the presence of 20 U/ml DNase I and 1 mg/ml lysozyme. Cells were lysed by three rounds of freeze and thaw (30 min freezing in −80°C and complete thawing in ice water). Ten A_260_ units was layered onto 5–45% (*w*/*v*) sucrose gradient for polysome profiling. The 5% and 45% (*v*/*v*) sucrose solutions were prepared by dissolving sucrose in overlay buffer (10 mM HEPES pH 7.5, 50 mM KCl, 10 mM NH_4_Cl, 10.25 mM MgOAc, and 0.25 mM EDTA pH 8.0), and 6 mM β-ME was added before use. The gradients were formed using Gradient Master (BioComp, Munich, Germany). The sucrose gradients were centrifuged at 36,000 RPM for 1.5 h (ω^2^*t* = 7.6746 × 10^10^) at 4°C using SW 41 Ti rotor (Beckman Coulter, Brea, CA, United States). A density gradient fractionator system (Brandel, Gaithersburg, MD, United States) was used to analyze the ribosomal particles by continuous monitoring of A_260_ and to fractionate the samples.

### Tandem Mass Tag-Mass Spectrometry-Based Quantitative Proteomics

Tandem mass tag-mass spectrometry (TMT-MS) was carried out as described in a previous work (Park et al., 2019), with slight modifications. K12WT, Δ*bipA*, Δ*bipA* (pCA24N-BipA), Δ*rluC*, and Δ*rluC/*Δ*bipA* were cultured in 200 ml LB at 25°C in a shaking incubator at 200 RPM to OD_600_ ≈ 1.0. The cultures were immediately cooled on ice before centrifugation at 4,500 RPM for 8 min at 4°C. The resulting cell pellets were resuspended in 1 ml TMT lysis buffer [100 mM tetraethylammonium bromide (TEAB) pH 8.5, 1 mM PMSF, 1% Triton X-100, and EDTA-free protease inhibitor cocktail]. Lysis was done using sonication for 5 min at 40% amplitude with pulses set at 5 s on followed by 5 s off. Lysates were incubated at 4°C on a rotator for 1 h and then clarified by centrifugation at 15,000 RPM for 45 min at 4°C. The clarified lysates were filtered through a 0.22-μm spin filter (Corning^®^ Costar^®^ Spin-X^®^) and sent to the Proteomic Core Facility of the Biological Research Center of Nanyang Technological University (Singapore) for TMT-MS services. The TMT-MS was done with technical triplicates.

### β-Galactosidase Assay

The β-galactosidase assay was performed using 96-well microplates by referring to a reported work (Schaefer et al., 2016). The *obgE*:: pVIK111 and *deaD*:: pVIK111 strains were cultured in 10 ml LB at 25°C with shaking at 200 RPM. When the OD_600_ reached the value of 0.2, 0.5, and 1.0, 20 μl of the culture was collected and mixed with 80 μl permeabilization solution (100 mM Na_2_HPO_4_, 20 mM KCl, 2 mM MgSO_4_, 0.04% sodium deoxycholate, 5 mM β-mercaptoethanol, and 1 mg/ml lysozyme) in a 96-well microplate (Nunc^TM^ MicroWell^TM^, Thermo Scientific, Waltham, MA, United States). Permeabilizing samples were stored at 4°C until all the samples were prepared, and then, 25 μl of permeabilized samples were mixed with 150 μl of substrate solution (60 mM Na_2_HPO_4_, 40 mM NaH_2_PO_4_, 1 mg/ml ONPG, and 5 mM β-mercaptoethanol) in a 96-well microplate (Nunc^TM^ MicroWell^TM^, Thermo Scientific, Waltham, MA, United States) and mixed well before loading the plate without its lid into the TECAN Spark^TM^ 10M multimode microplate reader. The OD_420_ absorbance was measured every 5 min for a total of 80 min incubation at 37°C. The settings for the OD_420_ absorbance measurement were 25 flashes and 120-ms wait time between wells. Biological triplicates were analyzed for all strains.

### Purification of Pre-50S Particles

*E. coli* Δ*bipA* strain was cultured in large scale (six flasks of 800 ml LB) at 25°C with shaking at 200 RPM, until the OD_600_ ≈ 0.4–0.5. The cultures were immediately cooled down on ice before centrifugation at 4,000 RPM for 15 min using JLA-8.1000 rotor (Beckman Coulter, Brea, CA, United States). The resulting pellets were pooled and resuspended in 30 ml lysis buffer (20 mM HEPES pH 7.5, 10.5 mM MgOAc, 100 mM NH_4_Cl, 0.5 mM EDTA pH 8.0, 6 mM β-ME, 20 U/ml DNase I, and 1 mg/ml lysozyme) and aliquoted into Eppendorf tubes to go through three rounds of freeze and thaw for cell lysis. The lysates were centrifuged at 14,500 RPM for 30 min at 4°C, and the supernatants were pooled. The supernatant was diluted using lysis buffer to a concentration of 80 A_260_ units in a final volume of 800 μl. Then, 10–25% (*w*/*w*) sucrose gradients were formed by mixing sucrose solution with overlay buffer (10 mM HEPES pH 7.5, 50 mM KCl, 10 mM NH_4_Cl, 10.2 mM MgOAc, and 0.25 mM EDTA pH 8.0). Onto the sucrose gradients, 800 μl of samples were layered in 38.5-ml polyallomer open-top tubes (Beckman Coulter, Brea, CA, United States). The gradients were centrifuged at 19,000 RPM for 17.5 h (ω^2^*t* = 2.5 × 10^11^ at 4°C) in SW 28 Ti rotor (Beckman Coulter, Brea, CA, United States). Ribosomal particles were analyzed by a density gradient fractionator system (Brandel, Gaithersburg, MD, United States) by continuous monitoring of A_260_ and to fractionate the samples. Fractions that corresponded to the peak between the 30S and 50S subunit peaks were collected and pooled. Pooled samples were split into 26-ml polycarbonate bottles with cap assembly (Beckman Coulter, Brea, CA, United States) and centrifuged at 43,000 RPM for a minimum of 19 h at 4°C in Ti70 rotors. The supernatant was discarded, and the resulting pellets were washed with 70S buffer (5 mM HEPES pH 7.5, 50 mM KCl, 10 mM NH_4_Cl, and 10 mM MgOAc) twice to remove the sucrose. The pellets were resuspended in 1 ml of 70S buffer and stored at −80°C.

### Purification of BipA

BipA was overexpressed from the plasmid pNIC28-Bsa-*bipA* in *E. coli* BL21 (DE3) and purified through three consecutive chromatography steps. Cells transformed with the plasmid were cultured large scale at 37°C until the OD_600_ ≈ 0.8 and then overexpression was induced by adding isopropyl β-D-thiogalactoside (IPTG) to a final concentration of 125 μM. Culture temperature was reduced to 16°C upon IPTG induction for overnight incubation. Cells were harvested by centrifugation at 4,000 RPM for 15 min at 4°C. The resulting cell pellet was resuspended in 100 ml of lysis buffer [50 mM NaH_2_PO_4_ pH 8.0, 300 mM NaCl, 10% (*v*/*v*) glycerol, and 5 mM β-ME] and lysed using LM20 Microfluidizer^®^ (Microfluidics^TM^, Newton, MA, United States) with 20,000 PSI pressure. The lysate was clarified by centrifugation at 20,000 RPM for 1 h at 4°C using a JA-25.50 rotor, filtered through a 0.45-μm filter, and kept on ice before loading onto HisTrap^®^ HP 5 ml Ni^2+^ column for affinity chromatography using ÄKTA Purifier (GE Healthcare Life Sciences, Marlborough, MA, United States). The column was then washed with two column volumes (CV) of lysis buffer. The elution was performed by gradually increasing the concentration of His elution buffer [50 mM NaH_2_PO_4_ pH 8.0, 300 mM NaCl, 10% (*v*/*v*) glycerol, 5 mM β-ME, and 500 mM imidazole). The fractions containing BipA as determined by sodium dodecyl sulfate–polyacrylamide gel electrophoresis (SDS-PAGE) were pooled and diluted with 25 mM Tris pH 8.0 buffer to a final concentration of 50 mM NaCl before loading onto HiTrap^®^ Q Fastflow 5-ml column for anion exchange chromatography using ÄKTA Purifier. The column was then washed with two CVs of Q buffer A (25 mM Tris pH 8.0, 50 mM NaCl, and 5 mM β-ME). The elution was carried out by gradually increasing the concentration of Q buffer B (25 mM Tris pH 8.0, 1 M NaCl, and 5 mM β-ME). The fractions containing BipA as determined by SDS-PAGE were pooled and concentrated to a volume of 5 ml using Amicon^®^ Ultra-15 Centrifugal Filter Units with 30-kDa cut-off membrane (MERCK, Darmstadt, Germany). A concentrated sample was loaded onto HiLoad^®^ 16/60 Superdex^®^ 200-pg column equilibrated with GF buffer (25 mM Tris pH 8.0, 100 mM NaCl, and 5 mM β-ME) for size exclusion chromatography using ÄKTA Explorer. The fractions containing BipA were pooled and concentrated to 13 mg/ml using Amicon^®^ Ultra-15 Centrifugal Filter Units with 10-kDa cut-off membrane (MERCK, Darmstadt, Germany). The concentrated proteins were snap frozen using liquid nitrogen and stored at −80°C.

### Ribosome Binding Assay

For at least 30 min on ice, 50 μl of BipA (13 mg/ml) in GF buffer was incubated with 100 μM GDPCP. For western blotting of polysome profiling fractions, 10 A_260_ units of Δ*bipA* cell lysate was prepared, and BipA pre-incubated with GDPCP was added five times in excess (10 A_260_ = 23.9 nM; BipA concentration = 1.195 μM) and incubated on ice for 1.5 h. The sample was layered onto 5%–45% sucrose gradient in overlay buffer (10 mM HEPES pH 7.5, 50 mM KCl, 10 mM NH_4_Cl, 10.25 mM MgOAc, and 0.25 mM EDTA pH 8) in 13.2-ml thin-wall polypropylene tubes (Beckman Coulter, Brea, CA, United States) and centrifuged at 36,000 RPM for 1.5 h (ω^2^*t* = 7.6746 × 10^10^) at 4°C using an SW 41 Ti rotor (Beckman Coulter, Brea, CA, United States). The gradients were fractionated by 10 drops per 1.5-ml Eppendorf tubes. Fractions were precipitated by adding 2.5 times sample volume of ice-cold 100% ethanol and one-tenth sample volume of 3 M NaOAc and incubation at −20°C overnight. The precipitated ribosomal samples were recovered by centrifuging the samples at 14,500 RPM for 30 min at 4°C, then air-dried before resuspending in 10 μl of RNase-free water. Recovered ribosomal samples were then loaded onto 10% polyacrylamide gel followed by semi-dry transfer onto nitrocellulose membrane. Recombinant BipA was detected by western blotting using 1:2,000 of HRP-conjugated anti-His_6_ antibody (Santa Cruz Biotechnology, Dallas, TX, United States), and then, Clarity ECL western blotting substrates (Bio-Rad, Hercules, CA, United States) was applied before visualization using ChemiDoc^TM^ (Bio-Rad, Hercules, CA, United States).

BipA binding was also analyzed by co-pelleting through sucrose cushion. Two separate samples were prepared on ice. Then, 50 μl of BipA (13 mg/ml) in GF buffer was incubated with 100 μM GDPCP for at least 30 min on ice. The sample with BipA pre-incubated with GDPCP only was prepared by mixing 1X Buffer G (5 mM HEPES pH 7.5, 50 mM KCl, 10 mM NH_4_Cl, 10 mM MgOAc, and 6 mM β-ME) and 144 μM of BipA pre-incubated with GDPCP and topped up to 50 μl with RNase-free water. The BipA pre-incubated with GDPCP complex with pre-50S particles was prepared by mixing 1X Buffer G with 144 μM of BipA pre-incubated with GDPCP and 24 OD_260_ units (0.576 μM) of pre-50S particles and then topped up to 50 μl with RNase-free water. Then, samples were layered onto 1.1 M sucrose in 1X Buffer G and centrifuged at 45,000 RPM in a TLA-100 rotor for 16 h at 4°C. Post-centrifugation, 1 μl of supernatant was aliquoted from each sample. The remaining supernatant was discarded, and the pellet was washed with 1X Buffer G three times to remove the sucrose. The pellets were then resuspended in 20 μl of 1X Buffer G. The RNA concentration of sample with BipA pre-incubated with GDPCP complex with pre-50S particles was measured using a NanoDrop 2000c spectrophotometer (Thermo Fisher Scientific, Waltham, MA, United States) and adjusted to 24 OD_260_ units using 1X Buffer G if dilution was required. Samples for PAGE were prepared by mixing 1 μl of the sample, 2.5 μl of 4X loading buffer, 1 μl of β-mercaptoethanol, and 5.5 μl of RNase-free water and incubated at 70°C for 10 min. Then, samples were loaded onto 4–12% NuPAGE Bis-Tris Gel (Invitrogen, Waltham, MA, United States) and run in 1X MES buffer at 200 V.

## Results

### Deletion of Genomic *bipA* Gene Affects *E. coli* Growth at Suboptimal Temperature

**FIGURE 1:**
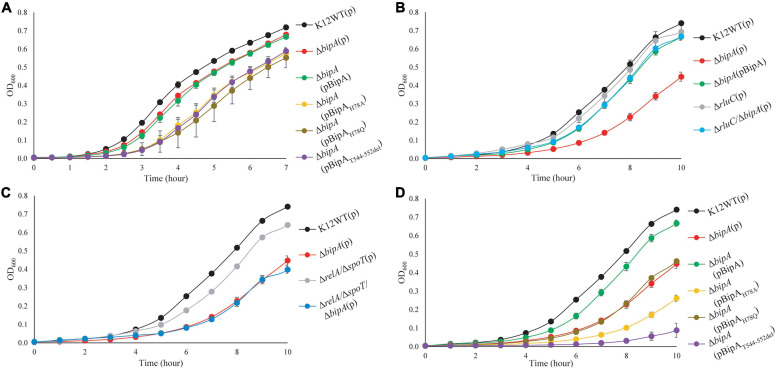
Growth curves of *E. coli* wild-type, *bipA*-deficient, and complementation strains under optimal (37°C) and suboptimal (25°C) growth temperatures. **(A)** Growth curves at 37°C of *E. coli* with various BipA mutations. Under optimal condition, the strain Δ*bipA* did not present a significant growth defect, but the strains Δ*bipA* transformed with pCA24N-BipA variants presented a significant delay in growth. **(B)** Growth curves at 25°C of *E. coli* with deletions for *bipA* and/or *rluC*. Loss of *bipA* (Δ*bipA*) led to growth defect at 25°C, which can be complemented by the presence of BipA expression (pCA24N-BipA) or suppressed by genomic deletion of *rluC*. The complementation was judged by the growth curve progression of the diverse strains (Δ*bipA*, Δ*bipA* + pCA24N-BipA, Δ*rluC*, and Δ*bipA* + Δ*rluC*) in comparison to the wild-type strain (K12WT). **(C)** Growth curves at 25°C of *E. coli* with deletions for *bipA*, relA, and/or spoT. Deletion of both *relA* and *spoT* caused a slight growth delay but did not exacerbate the growth delay of Δ*bipA* in the triple knock-out strains. **(D)** Growth curves at 25°C of *E. coli* with various BipA mutations. The Δ*bipA* strain expressing pCA24N-BipA variants presented different growth curves at 25°C. The cells with BipA_H__7__8Q_ demonstrated the same growth rate as the strain Δ*bipA* (pCA24N), while preceding with BipA_H__7__8A_ that preceded with BipA_T__544___*D*__55__2d__el_. Note that biological triplicates were analyzed, and pCA24N without gene of interest was transformed into cells not harboring any plasmid so that all strains have the similar cellular burden of holding a plasmid. The “p” in the bracket alone or preceding “BipA” represents plasmid pCA24N.

While the growth defects of *E. coli* Δ*bipA* knock-out strains at suboptimal culturing temperature (mostly at 20°C) have been reported by several groups (Pfennig and Flower, 2001; Krishnan and Flower, 2008; Choudhury and Flower, 2015; Choi and Hwang, 2018), the phenotype is not well consistent and understood. For a better understanding of the importance of BipA in cold stress conditions, we first determined the growth curves of *E. coli* K12 strain (K12WT) and its corresponding *bipA* knock-out strain (Δ*bipA*), both harboring the empty pCA24N vector, under optimal (37°C, Figure 1A) and suboptimal (25°C, Figure 1B) temperatures. While the two strains demonstrated a similar growth rate at 37°C (Figure 1A), the Δ*bipA* strain revealed a notable growth retardation at 25°C, resulting from a significantly longer lag phase (Figure 1B). Further corroborating the role of BipA in cold stress is the finding that BipA expression from plasmid pCA24N-BipA could restore the growth of Δ*bipA* strain, albeit not entirely (Figure 1B). In line with a previous report (Choudhury and Flower, 2015), RluC deficiency (Δ*rluC*) can complement the growth defect of Δ*bipA* at 25°C (Figure 1B).

Next, we sought to investigate the effect of the loss of genes linked to bacterial stress response. Namely, we examined whether the growth defect is exacerbated if alarmone synthetase genes *relA* and *spoT* were deleted in the Δ*bipA* background. Our results showed that the double mutant strains (Δ*relA*/Δ*spoT*) demonstrated a slight growth defect compared to the wild type, whereas the growth curve was similar to *relA*/*spoT* knock-out in the Δ*bipA* background (triple mutations) (Figure 1C), implying that alarmone level perhaps has little influence on the growth of *E. coli* at suboptimal temperature.

GTP hydrolysis activity is important for trGTPase turnover on ribosome and in turn its physiological function through conformational change (Kumar et al., 2015; Ero et al., 2016). To study the significance of GTP hydrolysis for BipA functioning under sub-optimal growth temperature, mutations were introduced into the plasmid-borne *bipA* gene and subsequently transferred to the Δ*bipA* strain. We mutated the proposed catalytic residue histidine 78 to alanine (H78A) or glutamine (H78Q) to abolish the GTP hydrolysis, respectively, based on structure and sequence comparison of BipA with other trGTPases such as EF-G and EF-Tu (Scarano I, Krab et al., 1995; Gao et al., 2009; Schmeing et al., 2009; Koripella et al., 2015). In addition, the C-terminal loop (CTL) of BipA was truncated (T544_D552del) given that the CTL is believed to be essential for BipA binding with 70S ribosome (deLivron et al., 2009; Kumar et al., 2015). Growth complementation results showed that under an optimal growth condition (37°C), leaky expression of BipA mutants BipA_H__7__8A_, BipA_H__7__8Q_, and BipA_T__544___*D*__55__2d__el_ would have a negative effect on the growth of Δ*bipA* strain whereas the native BipA expression had no effect on cell growth (Figure 1A). In line with the role of GTP hydrolysis essential for BipA turnover, these findings likely suggest that the aforementioned mutations cause BipA turnover defects, resulting in the “trapped” ribosomes leading to decrease in translation and, ultimately, affecting cell growth. By being “trapped,” the ribosomes would be prevented from carrying out its task due to the bound translational factors being unable to dissociate itself from the ribosome, similar to that in which EF-G was trapped by fusidic acid (Gao et al., 2009).

Interestingly, the growth defects that vary in magnitude were observed when the Δ*bipA* strains transformed with diverse *bipA* mutations were grown at suboptimal temperature of 25°C (Figure 1D). Namely, the growth complementation of Δ*bipA* strain by plasmid-borne BipA was not achieved for BipA_H__7__8A_, BipA_H__7__8Q_, and BipA_T__544___*D*__55__2d__el_ mutants, whereas BipA could completely restore the growth of Δ*bipA* strain (Figure 1D). In particular, the expression of BipA_H__7__8A_ and BipA_T__544___*D*__55__2d__el_ mutants resulted in further notable defects in Δ*bipA* strain at suboptimal conditions, suggesting that ribosome binding and GTP hydrolysis are crucial for the role of BipA in bacterial growth under cold shock stress.

Taken together, our results demonstrated that the elimination of alarmone synthesis by removing RSH proteins has a minute effect on cell growth of at low temperature, whereas the loss of *bipA* would cause a significant growth defect, which could be complemented or suppressed by pCA24N-BipA supplementation and genomic *rluC* deletion, respectively.

### Loss of *bipA* Gene Causes Swimming Motility Defect in *E. coli* at Suboptimal Temperature

**FIGURE 2:**
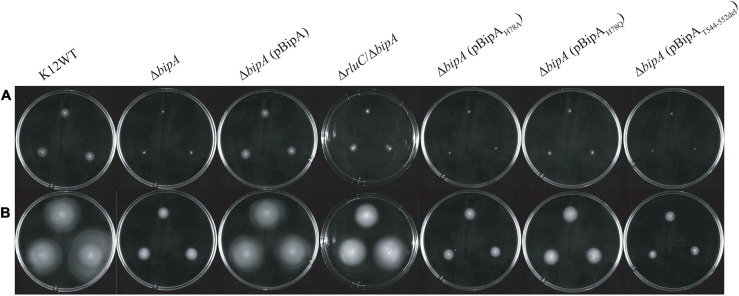
The effect of BipA on swimming motilities of various strains of *E. coli* K12 BW25113 after **(A)** 24-h incubation and **(B)** 48-h incubation. The agar plate-based assay was employed, and it was assessed by the development of chemotactic ring. **(A)** After 24-h incubation at room temperature (suboptimal temperature), cells with *bipA* deletion showed no notable chemotactic rings except for cells with double mutations (Δ*rluC*/Δ*bipA*). Cells with the plasmid-borne BipA were compensated for the loss of genomic *bipA* and yielded chemotactic ring with similar size to the strain K12WT. The Δ*bipA* strains expressing BipA mutants also demonstrated the lack of chemotactic ring except for the strain Δ*bipA* (pCA24N-BipA_H__7__8Q_), which showed slight development of chemotactic ring as compared to the Δ*bipA* strain. **(B)** After 48-h incubation, all the strains developed chemotactic rings despite variations in size. The Δ*bipA* strain expressing BipA mutants presented significant increase in chemotactic ring size, where the strain Δ*bipA* (pCA24N-BipA_H__7__8Q_) yielded chemotactic ring larger than the strains Δ*bipA* (pCA24N-BipA_H__7__8A_ and pCA24N-BipA_T__544___*D*__55__2d__el_). The “p” in the bracket alone or preceding “BipA” represents plasmid pCA24N.

It was recently reported that *E. coli* with *bipA* deletion demonstrated motility defects while incubated at 20°C (Choi and Hwang, 2018). Hence, we would like to examine the swimming motility of our *E. coli* strains using agar plate assay and incubation at room temperature, with the agar plate images after 24- and 48-h incubation shown (Figures 2A,B). The results of 24-h incubation demonstrated that the swimming motility was severely diminished for Δ*bipA* strain, and it could be complemented by plasmid harboring native *bipA* gene, but not the *bipA*_H__7__8A_, *bipA*_H__7__8Q_, and *bipA*_T__544___*D*__55__2d__el_ mutants (Figure 2A). Interestingly, all Δ*bipA* strains produced chemotactic rings after 48-h incubation (Figure 2B). Despite that the rings for the BipA mutants are notably smaller than those for the strains expressing BipA, the results clearly showed that swimming motility was not diminished, but rather reduced, for Δ*bipA* and the *bipA* mutants. Furthermore, Δ*bipA* strain expressing BipA_H__7__8Q_ produced significantly larger rings than Δ*bipA* strain expressing BipA_H__7__8A_ or BipA_T__544___*D*__55__2d__el_ after 48-h incubations (Figure 2B), which suggests that H78Q substitution retains BipA function to a certain extent, likely the ribosome binding and transition state stabilizing. In line with this hypothesis, Δ*bipA* (pCA24N-BipA_H__7__8A_) produced a significantly larger ring than Δ*bipA* (pCA24N-BipA_T__544___*D*__55__2d__el_) after 48-h incubation, indicating that the truncation of BipA CTL causes more severe motility defects for *E. coli* (Figure 2B), and note that BipA CTL is required for BipA binding to ribosome (deLivron et al., 2009; Kumar et al., 2015). On the other hand, while RluC-deficient (Δ*rluC*) strain behaved similarly to *E. coli* wild-type strain (K12WT), the Δ*rluC*/Δ*bipA* strain demonstrated significant suppression on the swimming defect of Δ*bipA* (Figures 2A,B); this revealed a functional link between BipA and RluC as previously reported (deLivron et al., 2009).

Agar plate assay alone does not provide sufficient evidence to conclude whether the chemotactic ring represents the swimming motility of the bacteria, given that the possible influence of cell growth on the ring formation cannot be completely ruled out. The rings observed on semi-solid agar might represent bacterial growth instead of swimming motility because all the strains except K12WT and Δ*bipA* (pCA24N-BipA) did not present the typical chemotactic rings where highly motile populations form an outer layer of the ring (Koster et al., 2012; Cremer et al., 2019; Liu et al., 2019). Furthermore, the cells might be defective in swimming and were tumbling, or the observations were restricted to semi-solid environment (Kinosita et al., 2020). Thus, inverted microscopy was employed to detect the diluted overnight cultures of K12WT, Δ*bipA*, Δ*bipA* (pCA24N-BipA), and Δ*rluC*/Δ*bipA* cells (Supplementary Movies 1–4), and these animated movies show that all four strains demonstrated swimming motility in liquid media. Taken together, strains with *bipA* yielded larger chemotactic rings at room temperature, and deletion of *bipA* caused a significant delay in the appearance of chemotactic rings, which could be complemented by introducing functional BipA or suppressed by genomic deletion of *rluC*.

### The Effect of BipA Mutations on the Defect of Ribosome Assembly

**FIGURE 3:**
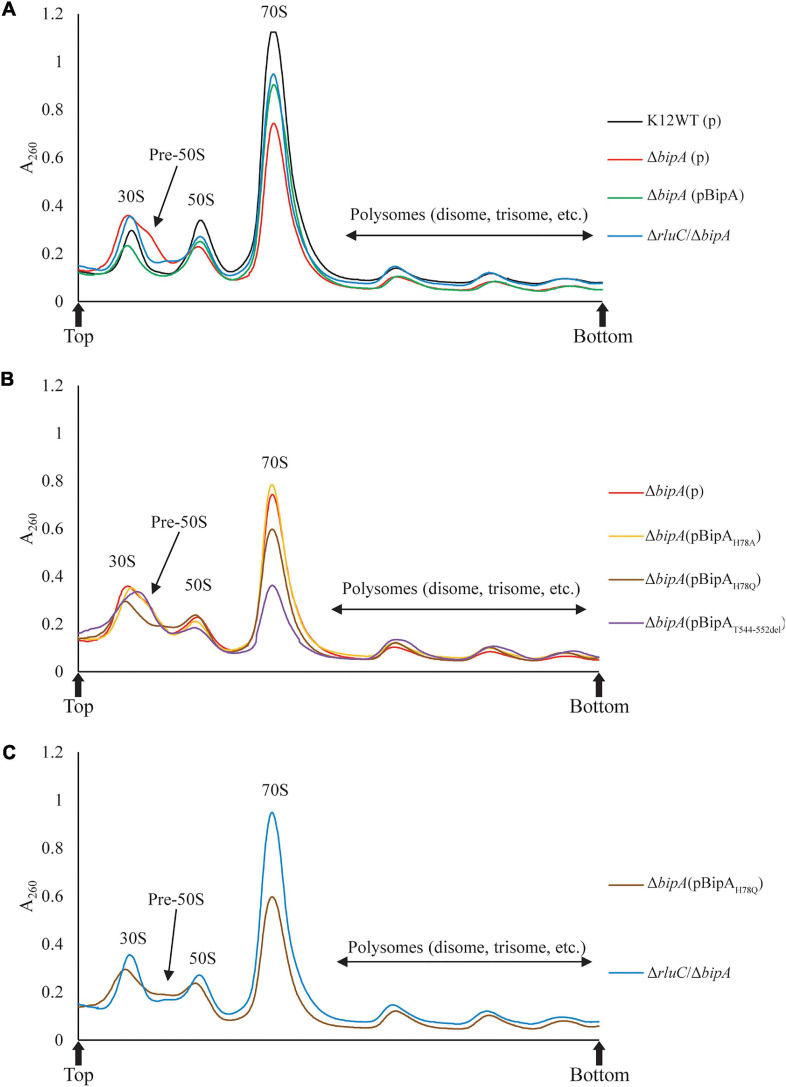
Ribosomal particle distribution showing ribosome assembly defects caused by *bipA* deletion and BipA mutants. Peaks corresponding to polysomes, 70S ribosome, and free subunits are indicated. **(A)** Using K12WT (black) as a reference, Δ*bipA* (red) presented accumulation of ribosomal subunits and reduction of 50S and 70S ribosomal particles, deduced from the higher 30S peak and lower 50S and 70S peaks, respectively. The pre-50S peak appeared as a minor peak between 30S and 50S peaks in Δ*bipA* ribosomal particle distribution. Although with a lower 70S peak, the profile of Δ*bipA* (pCA24N-BipA) (green) was similar to K12WT. The Δ*rluC*/Δ*bipA* (blue) yielded similar 50S and 70S peaks as K12WT, but similar 30S peak as Δ*bipA*. Notably, the region between the peak of 30S and 50S subunits was slightly elevated indicating maturing pre-50S. **(B)** The Δ*bipA* (pCA24N-BipA_H__7__8A_) (yellow) yielded almost identical ribosomal particle distribution as Δ*bipA*. While Δ*bipA* (pCA24N-BipA_H__7__8Q_) (brown) had lesser 70S ribosome, the pre-50S peak was not visible. The Δ*bipA* (pCA24N-BipA_T__544___*D*__55__2d__el_) (purple) produced the least 70S and 50S particles with a skewed 30S peak, which may include a large population of pre-50S particles. **(C)** A comparison between sucrose gradient profiles of the strains Δ*rluC*/Δ*bipA* and Δ*bipA* (pCA24N-BipA_H__7__8Q_). The data showed similarity in terms of reduced pre-50S with elevated area under the peak between 30S and 50S peaks, likely representing a population of pre-50S that had mature further than what was seen in Δ*bipA*. The “p” in the bracket alone or preceding “BipA” represents plasmid pCA24N. Peaks corresponding to subunits (30S, pre-50S, and 50S), monosomes (70S), and polysomes are indicated. Top and bottom of each gradient are marked with arrows.

The loss of *bipA* had been found to cause ribosome assembly defect with the accumulation of pre-50S, which can be alleviated by expressing BipA from a plasmid or genomic deletion of *rluC* (Krishnan and Flower, 2008). Very recently, BipA has been implicated in ribosome (specifically large subunit) assembly at low temperature growth (Choi and Hwang, 2018; Gibbs et al., 2020). Here, we examine whether the *bipA*_H__7__8A_, *bipA*_H__7__8Q_, and *bipA*_T__544___*D*__55__2d__el_ mutants affect the complementation of ribosome assembly defect in the Δ*bipA* cells through sucrose gradient sedimentation analysis. As shown in Figure 3A, compared to the wild-type strain K12WT, *bipA* deficiency resulted in significantly reduced 70S ribosome and 50S subunit populations (Figure 3A), and simultaneously, a minor peak between the 30S and 50S peaks was observed, which is likely representing a population of pre-50S particles and is consistent with the previous results (Choi and Hwang, 2018; Gibbs et al., 2020). Similar to that presented by Gibbs et al. (2020), the pre-50S peak was not observed in the ribosomal particle distribution of Δ*bipA* expressing exogenous BipA (pCA24N-BipA), suggesting that BipA is involved in ribosome assembly, particularly in the maturation of the 50S subunit (Figure 3A). On the other hand, Δ*bipA* strain with *rluC* genomic deletion (Δ*rluC*/Δ*bipA*) yielded ribosomal particle distribution without a notable pre-50S peak, which differs from the previous study (Choudhury and Flower, 2015), and concurrent *rluC* deletion failed to fully compensate the ribosome assembly defect of *bipA* deficiency (Figure 3A). Note that all the strains demonstrated a similar profile of polysome level.

Comparisons among the Δ*bipA* strains expressing pCA24N-BipA mutants revealed very interesting results on the ribosomal particle distribution (Figure 3B). The expression of BipA_H__7__8A_ in the Δ*bipA* strain resulted in a very similar profile to that of the control (Δ*bipA* strain), suggesting that the H78A mutation might have rendered BipA non-functional (Figure 3B). In contrast, the ribosomal particle distribution of Δ*bipA* strain with the expression of BipA_T__544___*D*__55__2d__el_ showed the lowest 70S and 50S peaks of all strains as well as an abnormal 30S peak, demonstrating the importance of intact CTL (Figure 3B). The relatively skewed 30S peak might be an accumulation of heterogeneous population of ribosomal subunits and likely includes the pre-50S particles; therefore, the lower 50S peak perhaps resulted from fewer 50S subunits matured in the strain Δ*bipA* (pCA24N-BipA_T__544___*D*__55__2d__el_). The expression of BipA_H__7__8Q_ in the Δ*bipA* strain generated a medium level of compensation for BipA, demonstrated by the absence of pre-50S peak. In addition, the ribosomal particle distribution was significantly different from that of the Δ*bipA* (pCA24N-BipA_H__7__8A_) strain, but similar to that of the Δ*rluC*/Δ*bipA* strain, implying that H78Q substitution of BipA might have retained the function of BipA to a certain degree (Figures 3B,C). As *rluC* deletion is known to compensate the loss of BipA, the similarity in complementation would therefore support the notion that glutamine is able to partially substitute histidine as the catalytic residue of BipA GTPase activity (Koripella et al., 2015).

Taken together, ribosome assembly defect caused by the loss of endogenous BipA could be partially complemented by introducing BipA and the mutant BipA_H__7__8Q_ or suppressed by genomic *rluC* deletion, but not the BipA_H__7__8A_ and BipA_T__544___*D*__55__2d__el_, demonstrating varied and complicated effects of GTP hydrolysis and ribosome binding (e.g., CTL) of BipA in ribosome assembly at low temperature.

### Loss of *bipA* Resulted in Upregulation of Proteins Involved in RNA Metabolism

TMT is an isobaric mass tag-based multiplexed quantitative proteomics method by mass spectrometry (Thompson et al., 2003). Tryptic peptides from different samples are labeled with different isobaric tags for accurate relative quantitation of protein expression across the samples. Using tandem mass spectrometry, proteins can be identified by the fragment ions of peptides, and their expression levels quantitated with reported ion intensities. Next, we sought to further investigate the effect of BipA on protein expression and the rationale behind the suppression of Δ*bipA* phenotypes by genomic deletion of *rluC* for cells under suboptimal temperature with TMT approach. Volcano plots of the TMT proteomic datasets were used to determine significant changes in protein expression between different conditions. The cut-off for fold change (FC) with statistical significance (*p* < 0.05) was determined to be 1.5 (log_2_ abundance ratio = 0.585). Differentially expressed proteins were shortlisted for further data analysis and interpretation (Supplementary Tables 2–7). The data was reliable as validated by the protein expression level of the deleted genes.

**FIGURE 4:**
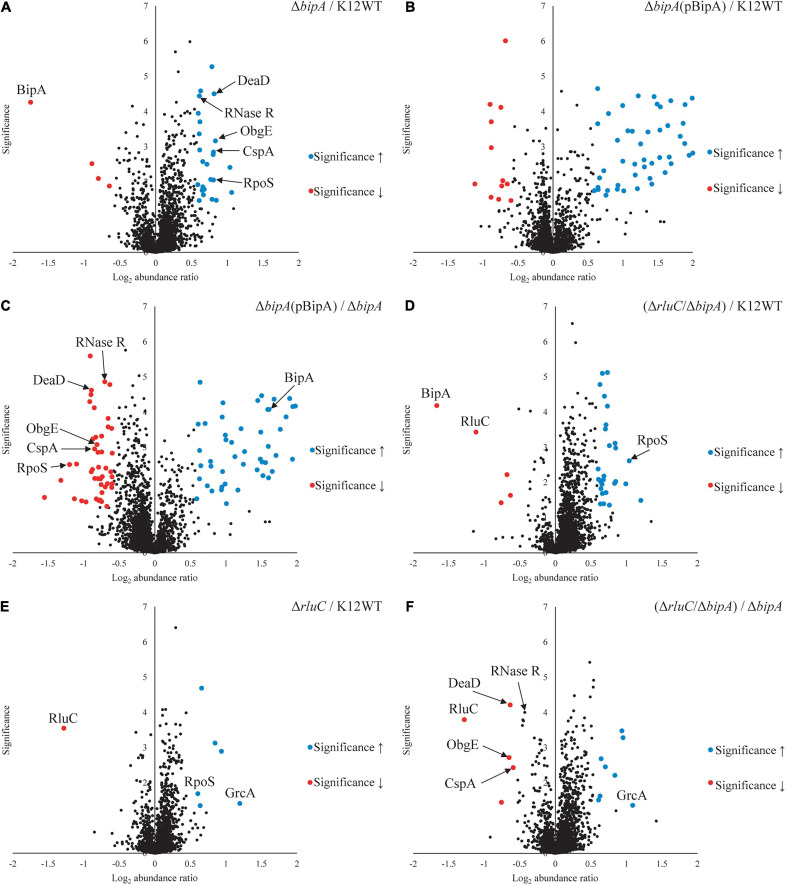
Tandem mass tag-mass spectrometry (TMT-MS) analysis of various strains of *E. coli* K12 BW25113. A TMT-based quantitative proteomic method was used to determine differential protein expression under suboptimal cell culture condition between wild-type, Δ*bipA* (pCA24N-BipA), and ΔrluC/Δ*bipA* strains. **(A)** A volcano plot showing protein expression level of the strain Δ*bipA* against the strain K12WT. The *bipA* was indeed deleted based on significantly low log_2_ FC. The five proteins DeaD, ObgE, RpoS, CspA, and RNase R yielded significantly higher reads in the strain Δ*bipA* than the strain K12WT. **(B)** The expression levels of DeaD, ObgE, RpoS, CspA, and RNase R proteins were not changed between K12WT and Δ*bipA* (pCA24N-BipA) strain. **(C)** Comparison of Δ*bipA* (pCA24N-BipA) against Δ*bipA*. We put DeaD, ObgE, RpoS, CspA, and RNase R in the negative log_2_ abundance ratio side of the plot, meaning peptide reads of the proteins were lesser in the presence of pCA24N-BipA. The blue dot labeled as BipA was an indication that BipA was indeed expressed. **(D)** The *rluC* deletion in Δ*bipA* background produced a volcano plot similar to **(A)**, but the DeaD, ObgE, CspA, and RNase R were close to K12WT as differential expressions against K12WT were not detected. As in **(A,D)**, higher readout of RpoS was detected in Δ*rluC*/Δ*bipA*. Red dots labeled with BipA and RluC showed that these *bipA* and *rluC* were indeed deleted. **(E)** Genomic deletion of *rluC* only yielded six differentially expressed proteins relative to K12WT, and out of which, two interesting changes were RpoS and GrcA upregulations. The significant negative log_2_ abundance ratio of RluC indicates that the gene was indeed removed. **(F)** Comparisons between Δ*rluC*/Δ*bipA* and Δ*bipA* showed a reduced expression level of the DeaD, ObgE, and CspA to wild-type level. The RNase R readout was found to be reduced by the loss of *rluC*, close to the cut-off for FC. The pBipA refers to pCA24N-BipA.

As compared to wild-type strain K12WT, several proteins demonstrated higher expression level in the Δ*bipA* strain in response to cold stress (Figure 4A). Particular attention was drawn to two proteins DeaD and ObgE given that both have been implicated in 50S subunit biogenesis (Charollais et al., 2004; Sato et al., 2005). Other proteins, with significantly higher expression level in Δ*bipA* but not implicated in ribosome assembly, include CspA, RNase R, and RpoS. In contrast, by introducing BipA (pCA24N-BipA) to the Δ*bipA* strain, the expression levels of DeaD, ObgE, CspA, RNase R, and RpoS become similar to those in the strain K12WT (Figure 4B). Furthermore, a comparison of expression levels of these five proteins in the Δ*bipA* (pCA24N-BipA) and Δ*bipA* strains showed lower levels for the former (Figure 4C). In addition, the proteins relevant to cell motility were found significantly upregulated while the Δ*bipA* strains express exogenous BipA (pCA24N-BipA) (Figures 4B,C and Supplementary Tables 3, 4), suggesting that BipA has a direct or indirect influence on bacterial motility. Notably, upregulation of these proteins also rationalized our motility assay for the role of BipA, as observed in Figure 2.

Furthermore, the expression levels of these proteins (except for RpoS) in the Δ*rluC*/Δ*bipA* double mutant strain were similar to those in the strain K12WT, within cut-off value for different expression level (Figure 4D). In the case of RpoS, it was significantly upregulated in the Δ*rluC* strain as compared to the strain K12WT (Figure 4E), implying that *rluC* genomic deletion would affect RpoS expression and thereby leading to an additional effect in the Δ*rluC*/Δ*bipA* strain. A comparison of protein expression level of the Δ*rluC*/Δ*bipA* strain with that of the Δ*bipA* strain also revealed significantly lower levels of DeaD, ObgE, and CspA (Figure 4F). Notably, the expression level of RNase R in the Δ*rluC*/Δ*bipA* strain was about 0.7 times lower than that in the Δ*bipA* strain with high statistical significance (log_2_ abundance ratio = 0.432), indicating that RNase R was indeed downregulated in Δ*rluC*/Δ*bipA*, but did not meet the FC cut-off. These observations together demonstrated that the upregulation of the aforementioned proteins likely was ascribed to compensation of the *bipA* loss in *E. coli* when cultured at suboptimal temperature.

Interestingly, the upregulation of GrcA, a stress-induced alternate pyruvate formate-lyase subunit, was observed in Δ*rluC* strains. The mRNA of GrcA can be cleaved by MazF, leading to leaderless mRNA with anti-Shine–Dalgarno sequence removed; therefore, the resultant mRNA is favorably translated by a ribosome (Vesper et al., 2011). The significance of GrcA in bacterial stress and cold shock response remains poorly understood, but it is of interest for further study.

### Ribosome Maturation Factor DeaD Is Upregulated in Δ*bipA* Strain at Suboptimal Growth Temperature

To further validate the upregulation of 50S biogenesis factors detected by TMT-MS, β-galactosidase reporter assay was employed. The *lacZ* gene was inserted downstream of the target gene in the genome, and its activity (β-galactosidase activity) could be easily measured. Therefore, a fusion design (target gene–*lacZ*) with the stop codon of the target gene excluded can be used to assess the expression level of this target protein based on the output of the β-galactosidase (LacZ) activity. In our case, the β-galactosidase activity was used to reflect the expression level of the fused DeaD (DeaD–LacZ) protein, which would be consequently used to validate our observation by TMT-MS.

**FIGURE 5:**
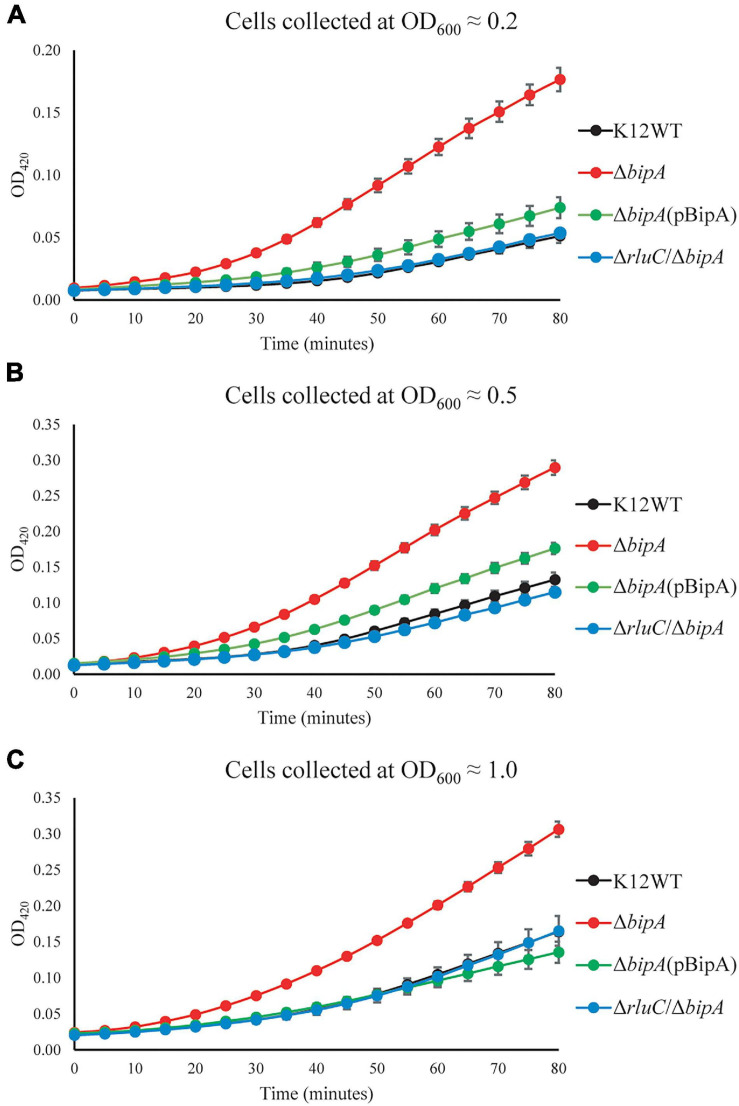
β-galactosidase activity assays demonstrated the upregulation of DeaD in the strain Δ*bipA*. The cells were harvested at OD_600_ ≈ 0.2 **(A)**, 0.5 **(B)**, and 1.0 **(C)**, respectively, and the activity was assessed by measuring the breakdown of ONPG through recording OD_420_. Δ*bipA* cells (red) harvested at all three OD_600_ presented the highest β-galactosidase activity, indicated by the significantly stronger OD_420_ absorbance over time as compared to K12WT (black), Δ*bipA* (pCA24N-BipA) (green), and Δ*rluC*/Δ*bipA* (blue). The pBipA refers to pCA24N-BipA.

Upon the *bipA* deletion (Δ*bipA*), the β-galactosidase activity was dramatically increased for all tests at OD_600_ ∼0.2, ∼0.5, and ∼1.0, demonstrating DeaD was highly expressed (Figures 5A–C). However, with further deletion of the gene *rluC* (Δ*rluC/*Δ*bipA*), the elevated β-galactosidase activity was reduced, almost to the level of the wild-type strain K12WT. Similarly, exogenous expression of BipA in its deletion strain (Δ*bipA* + pCA24N-BipA) was also able to complement the effect of *bipA* deletion on DeaD expression. Collectively, DeaD–LacZ activities for the three strains (K12WT, Δ*bipA* + pCA24N-BipA, and Δ*rluC*/Δ*bipA*) show similar time course, but lower than that for the *bipA* deletion strain (Δ*bipA*), suggesting that upregulation of DeaD was triggered by *bipA* deletion, which can be complemented or suppressed by overexpressing BipA or *rluC* genomic deletion, respectively. These data are in line with the expression changes in DeaD detected by TMT-MS and are in support of upregulated RNA helicase DeaD, which likely plays an important role in ribosome assembly at low temperature upon the loss of *bipA*. However, the precise and detailed mechanism remains unknown.

### BipA Binds Pre-50S Ribosomal Subunit and 70S Ribosome *in vitro*

**FIGURE 6:**
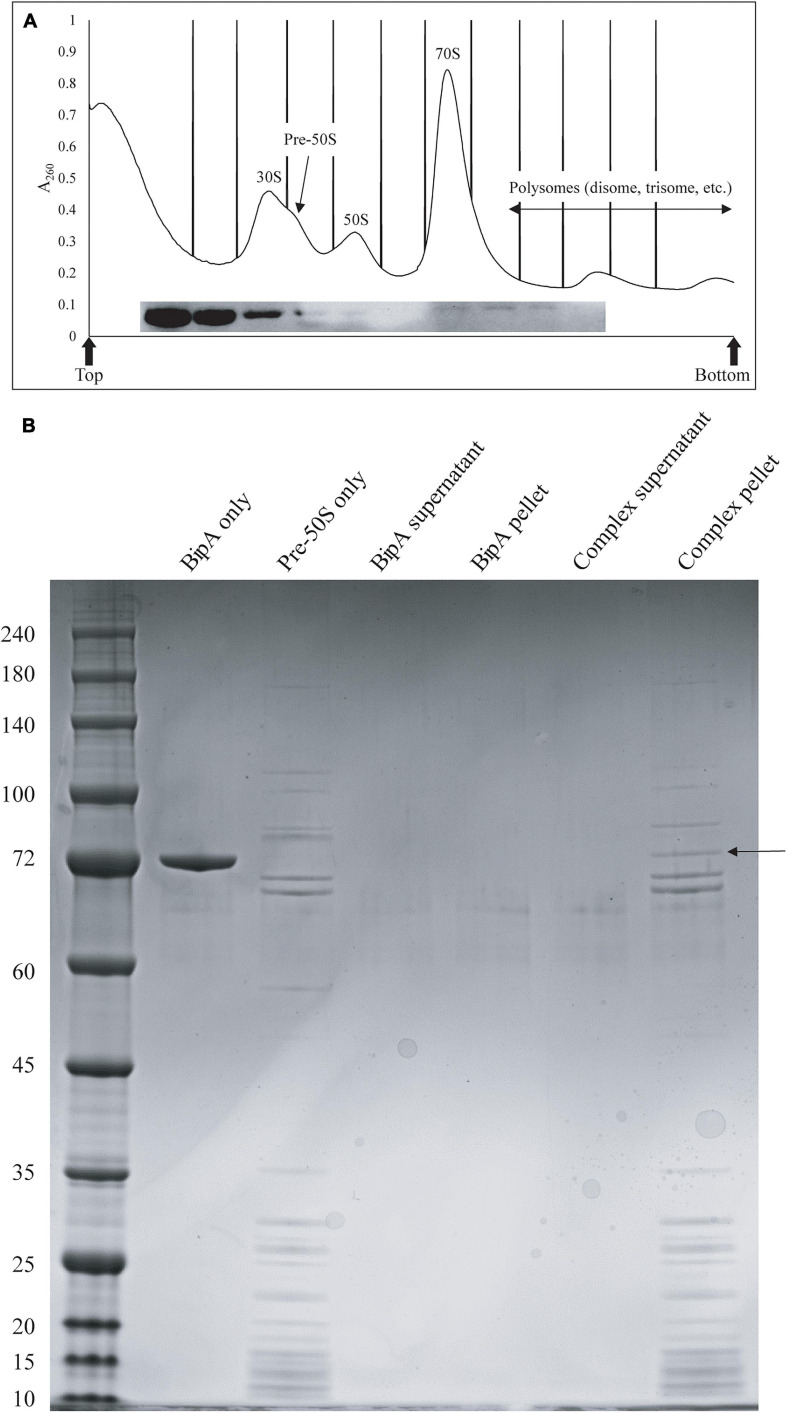
*In vitro* binding assays showed that BipA pre-incubated with 5X excess GDPCP bound to various ribosomal particles including pre-50S particles. **(A)** Ribosomal particle distribution of *in vitro* reconstitution of BipA-GDPCP with ribosomal particles. By analyzing the fractions from ribosomal particle distribution using western blot, most of the BipA pre-incubated with GDPCP were detected in the junk fractions before 30S peak, and the band intensity decreased from 30S toward polysomes. Peaks corresponding to subunits (30S, pre-50S, and 50S), monosomes (70S), and polysomes are indicated. Top and bottom of each gradient are marked with arrows. **(B)** SDS-PAGE check of ribosome fractions from **(A)**. BipA interaction with pre-50S was observed based on the presence of co-pelleting through sucrose cushion using ultracentrifugation. The band representing co-pelleted BipA is indicated by the black arrow in the “Complex pellet” lane, which consists of pelleted BipA pre-incubated with GDPCP-bound ribosome complex, suggesting that binding occurs.

We next tested whether BipA can bind pre-50S particle for its proposed function as 50S maturation factor through *in vitro* reconstitution. First, we mixed the purified BipA, which was pre-incubated with GDPCP with clarified lysate of the Δ*bipA* cells followed by sucrose gradient centrifugation and ribosome fractionation, and subsequently, western blot was employed to detect BipA. Our data clearly showed that BipA is able to co-sediment with both large and small ribosomal units, as well as the pre-50S ribosomal unit (Figure 6A). BipA could bind to even small ribosomal unit 30S, perhaps through its β-barrel domain II, implying a yet unknown function in bacterial translational machinery ribosome (Figure 6A). Second, we combined BipA pre-incubated with GDPCP and purified pre-50S population of Δ*bipA* strain, layered on top of 1.1 M sucrose cushion and performed high-speed centrifuge to find out if BipA would co-sediment with pre-50S. As shown in Figure 6B, the results clearly demonstrated that BipA was indeed co-sedimented with pre-50S, corroborating that BipA is capable of binding to the pre-50S ribosomal particle.

## Discussion

### GTP Hydrolysis and CTL of BipA Are Crucial for 50S Biogenesis at Low Temperature

The highly conserved *bipA* gene has been shown to be significant for bacterial growth and ribosome assembly at suboptimal temperature (Choudhury and Flower, 2015; Ero et al., 2016; Choi and Hwang, 2018; Gibbs et al., 2020). Here, we report that *bipA* deletion and mutagenesis significantly affect *E. coli* swimming motility (Figure 2). The experiment was carried out at room temperature (suboptimal for *E. coli* growth) and revealed that the swimming defect of the Δ*bipA* strain can be complemented or suppressed by the expression of plasmid-borne BipA (pCA24N-BipA) or the concurrent deletion of *rluC* gene (Δ*rlu*C/Δ*bipA*), respectively (Figure 2). The possible reason for this phenotype might be the decelerated global translation due to retardation of ribosome biogenesis, which subsequently shuts down energy-costly pathways, one of which involves the cell motility (Ottemann and Miller, 1997; Martínez-García et al., 2014). The observations of agar plate assay for these mutations correlate with the change of growth trend that can be seen in growth curves (Figure 1) as well as with ribosomal particle distribution observed in Figure 3, suggesting that the loss of *bipA* leads to a condition affecting growth, motility, ribosome assembly, and protein translation. Indeed, the ribosome assembly defect likely slows down protein translation as the concentrations of 70S ribosomes and polysomes are decreased (Peil et al., 2008; Gibbs et al., 2020). This retardation in protein translation then negatively affects the downstream processes including bacterial growth and motility (Rudra and Warner, 2004).

In this work, we showed that the phenotypes presented by the *bipA* deletion (Δ*bipA*) could be complemented by pCA24N-BipA or suppressed by *rluC* genomic deletion, suggesting that BipA is important for *E. coli* to thrive at suboptimal temperature, and pseudouridylation on the correct nucleotides (such as RluC involving U955, U2504, and U2580 of 23S rRNA) can be helpful for ribosome biogenesis in *E. coli* and therefore growth as well as motility. While there is no reported relation to bacterial motility, Choudhury and Flower (2015) proposed that suboptimal temperature caused the ribosome to be dependent on BipA for efficient assembly, and loss of 23S rRNA modification by RluC actually led to an alternative folding pathway that is BipA independent. This further suggests that the improvement of Δ*bipA* swimming is perhaps not motility regulation related. The unexpected appearance of the chemotactic rings for the strains Δ*bipA* and Δ*bipA* with plasmid-expressing BipA mutants after 48-h incubation suggests that the loss of *bipA* did not diminish swimming motility. Instead, the swimming was delayed probably due to the decreased rate of protein translation and cell growth caused by ribosome assembly defect. This is supported by the microscopy visualization of the bacteria cells grown overnight in liquid culture demonstrating their swimming motility (Supplementary Movies 1–4).

In this study, the plasmid-borne BipA, with H78A or H78Q mutation (catalytic residue H78) or CTL truncation (T544_D552del), was transformed into the Δ*bipA* strain to examine its effect on complementation for BipA, respectively. The rationale behind the mutations was based on previous mutagenesis studies on catalytic histidine of EF-Tu and EF-G (H84 and H91, respectively) and structural study where the CTL interacts directly with the A-loop of 50S subunit (Supplementary Figure 11). The H84Q substitution in EF-Tu showed a reduction of GTP hydrolysis by 35%, and H84A abolished the GTPase activity (Scarano I, Krab et al., 1995), while the activity of ribosome-associated GTP hydrolysis of EF-G with H91Q substitution was comparable to native protein, and it was found to be defective in organic phosphate release (Koripella et al., 2015). The finding that the expression of BipA_H__7__8A_ did not restore the ribosomal particle distribution as did the wild-type BipA was also observed in a recently published study where the expression of BipA_H__7__8A_ led to slow growth for both WT and Δ*bipA* strains (Gibbs et al., 2020). On the other hand, Gibbs et al. (2020) expressed suspicion that BipA_H__7__8A_ is able to bind 70S ribosome but is unable to facilitate GTP hydrolysis as well as the subsequent factor release, which ultimately led to halt in translation. The BipA_H__7__8Q_ presented interesting outcomes that could be summarized in three points: (1) similar growth curve as Δ*bipA* at suboptimal temperature despite longer lag phase at optimum condition (Figures 1C,D); (2) shorter lag phase than Δ*bipA* (pCA24N-BipA_H__7__8A_) and Δ*bipA* (pCA24N-BipA_T__544___*D*__55__2d__el_) at suboptimal temperature (Figure 1D); (3) similar ribosomal particle distribution as Δ*rluC*/Δ*bipA*, albeit with significantly fewer 70S ribosomal particles (Figure 3C). Based on the aforementioned examples of H84Q and H91Q, glutamine substitution might have rendered BipA less efficient in GTP hydrolysis and/or organic phosphate release (Scarano I, Krab et al., 1995; Koripella et al., 2015), leading to the retention on 70S ribosome for longer period of time and negatively impacting the assembly efficiency. Interestingly, BipA_T__544___*D*__55__2d__el_ seemed to have an inhibitory effect on *E. coli* growth and ribosome assembly at suboptimal temperature as deduced from the exacerbated growth defect as compared with the strain Δ*bipA* (Figure 1D) and lesser 50S and 70S observed in ribosomal particle distribution (Figure 3B). Kumar et al. (2015) reported that the region L543-E553 of CTL projects deep into the peptidyl transferase center (PTC), while the region N536-K542 is critical for ribosome binding. Therefore, the truncation of T544-D552 could potentially play a role in BipA association and function by establishing direct contact with the A-loop of 23S rRNA (Supplementary Figure 11). This corroborates with the study by deLivron et al. (2009) revealing that alanine substitutions of amino acids within the CTL hinder the binding of BipA to the 70S ribosome.

Collectively, growth curves, ribosomal particle distribution, and swimming motility assays demonstrated a correlation whereby the defect in ribosome assembly at suboptimal temperature leads to a delay in growth and swimming motility (Pfennig and Flower, 2001; Choudhury and Flower, 2015; Choi and Hwang, 2018). Perhaps, BipA is a ribosome biogenesis factor that is crucial for ribosome biogenesis at suboptimal temperature, as a previously reported role for BipA is incorporating the ribosomal protein L6 into the 50S ribosome (Choi and Hwang, 2018). In the recent study by Gibbs et al. (2020), structural block 3 of 50S ribosomal subunit demonstrated a growth condition-dependent assembly, including suboptimal temperature. While it was not evident that BipA has a direct role in delaying block 3 folding, the loss of *bipA* led to the accumulation of pre-50S without ribosomal protein L17, an r-protein associated with block 3 (Gibbs et al., 2020).

### Loss of *bipA* Leads to Upregulation of RNA Metabolism

TMT-MS revealed a significant upregulation of a number of proteins involved in RNA metabolism and chaperoning in the Δ*bipA* strain, but with expression comparable to the wild-type level in the strains Δ*bipA* (pCA24N-BipA) and Δ*rluC*/Δ*bipA*. Contrary to pCA24N-BipA complementation, the Δ*rluC/*Δ*bipA* strain presented an expression of majority of the proteins at above wild-type level while it suppressed Δ*bipA* phenotypes. Furthermore, the findings from TMT-MS seem to correlate with growth curve, ribosomal particle distribution, and swimming motility assays in a sense that significant changes observed in Δ*bipA* were found to be reversed in complementation strains. Furthermore, the proteins with significant changes in expression in the Δ*bipA* strains were involved in ribosome assembly, stress response, and growth. In particular, the expression of DeaD, ObgE, CspA, and RNase R was increased in the Δ*bipA* strain, but decreased in the complementation strains, indicating their involvement in the phenotypes of the Δ*bipA* strain. On the other hand, the expression of RpoS was higher in the absence of BipA expression as compared to K12WT, including the Δ*rluC*/Δ*bipA* strain. This observation can be explained as the influence of *rluC* deletion given that the RpoS in the Δ*rluC* strain was presented higher than the wild-type level, and thus, an additive effect of RpoS reads would be observed in the strain Δ*rluC*/Δ*bipA*. To date, no relationship between RluC and RpoS has been reported according to our knowledge, and it is an intriguing topic for further studies. Note that an increased sensitivity to several antibiotics caused by the inactivation of *rluC* has been reported (Murakami et al., 2005; Toh and Mankin, 2008). This is not surprising as the pseudouridines synthesized by RluC are situated in PTC (U955, U2504, and U2580) where a number of antibiotics bind; hence, the rluC deletion increased the antibiotic susceptibility, and *rpoS* could be upregulated as a stress response in the Δ*rluC* strain (Conrad et al., 1998).

Out of the five aforementioned proteins, DeaD and ObgE have been implicated in the biogenesis of ribosome large subunit, suggesting their upregulations may have a functional relationship with BipA *in vivo*. Similar to BipA, DeaD has been found to be associated with pre-50S particles (Charollais et al., 2004). DeaD has a role in rRNA structural rearrangement using its helicase activity, which aids in 50S biogenesis at low temperature (Charollais et al., 2004). Charollais et al. (2004) also demonstrated that the overexpression of DeaD was able to complement the growth defect of Δ*srmB* at low temperature. The finding that the DEAD-box RNA helicase SrmB facilitates 50S biogenesis during early maturation phase (Charollais et al., 2003) suggests an overlap of functions between DeaD and SrmB (Charollais et al., 2004). Such overlapping functions could be possible with BipA and DeaD as well, since both strains Δ*deaD* and Δ*bipA* showed accumulation of pre-50S, and Δ*deaD*/Δ*bipA* double knock-out produced an additive effect (Choudhury and Flower, 2015). From the study by Kitahara and Suzuki (2009), DeaD was shown to be likely to contribute to efficient assembly of circularly permuted rRNAs, implying that the cell would lose the ability to assemble rRNAs with scrambled domains in the absence of DeaD. This finding could be linked to a recent hypothesis on “limited parallel processing” of rRNA and serve as an explanation for the upregulation of DeaD upon the *bipA* deletion (Δ*bipA*), which is likely to be a strategy for *E. coli* to cope with the loss of *bipA* at suboptimal temperature (Davis et al., 2016). Davis et al. (2016) found that ribosome assembly is dynamic after observing pre-50S from L17-deficient cells mature into the 50S albeit at a slower rate, which the author considered a “limited parallel processing” (Davis et al., 2016). Such behavior confers the bacteria the flexibility in ribosome assembly should there be any factors that are undesirable for ribosome assembly. In addition, Davis et al. (2016) also found that 23S rRNA matures in the form of cooperative folding blocks, whereby different regions of 23S rRNA mature independently in parallel and come into contact with each other to form the tertiary structure. Collectively, their results indicate that ribosome maturation and assembly can occur in multiple pathways, demonstrating the flexibility of bacterial ribosome assembly when there is a bottleneck caused by the shortage of assembly factors or r-proteins.

The importance of RNA secondary structure destabilization at low temperature was demonstrated by Awano et al. (2007), where they presented the growth defect of Δ*deaD* at 15°C being complemented by the overexpression of CspA. The CspA is an RNA chaperone that is able to destabilize RNA secondary structure at low temperature with low substrate specificity (Phadtare, 2011). In addition, RNase R, the only 3′–5′ exonuclease in *E. coli*, is also able to complement the cold sensitivity of Δ*deaD*, indicating their tight relationship during cold shock (Awano et al., 2010). The RNase R consists of a helicase and an RNase domain that function independently, and its mutant with only helicase activity was able to complement the cold sensitivity of Δ*deaD*, revealing that the helicase activity was the key during cold shock (Awano et al., 2007). Notably, upregulation of RNase R by at least sevenfold is observed during cold shock in *E. coli* (Cairrão et al., 2003; Guan et al., 2005). Hence, the upregulation of DeaD, CspA, and RNase R in Δ*bipA* is probably an attempt by the cell to compensate the loss of BipA during 50S biogenesis in which BipA plays a yet unknown role.

In addition to the evidence presented in our work for BipA, the loss of various ribosome assembly factors results in the development of the common phenotype of cold sensitivity coupled with ribosome assembly defect. As summarized in Table 2, 10 out of 24 proteins have previously been reported to play a role in cold sensitivity when deleted or mutated (bold), and several others demonstrated involvement in cold shock. As shown in this study, the deletion of RluC suppresses the cold sensitivity of Δ*bipA*; the overexpression and deletion of RhlE complement the cold sensitivity of Δ*deaD* and Δ*srmB*, respectively (Jain, 2008); and LepA is released from the membrane during stress, including cold shock (Pech et al., 2011). Other than these, a recent report revealed that r-protein L6, RplF, an essential protein involved in ribosome biogenesis at low temperature, was missing from the pre-50S fraction of *E. coli* in the absence of *bipA* (Bosl and Böck, 1981; Shigeno et al., 2016; Choi and Hwang, 2018). These observations strengthen the connections among cold sensitivity, RNA metabolic process, ribosome biogenesis, and ribosome assembly factors and together support BipA as a *bona fide* ribosome assembly factor. In addition to the structure of BipA bound to 70S ribosome, it would be of interest to characterize the structure of BipA in complex with the intermediate state of ribosome (like pre-50S in Figure 3) during its biogenesis, which could offer atomic insights into how BipA facilitates ribosome assembly.

**TABLE 2:** Tabulated data adapted from Gibbs and Fredrick (2018), referring to proteins involved in ribosome assembly in *E. coli*; ObgE is included.

Assembly factor	Type	Ribosomal subunit involved
**RbfA**	**RNP**	**30S**
RimJ	RNP	30S
RimM	RNP	30S
RimP	RNP	30S
**YhbY**	**RNP**	**50S**
**KsgA**	**Modification enzyme**	**30S**
RsmC	Modification enzyme	30S
RlmA	Modification enzyme	50S
RlmE	Modification enzyme	50S
RluB	Modification enzyme	50S
RluC	Modification enzyme	50S
RluD	Modification enzyme	50S
**DeaD**	**Helicase**	**50S**
**DbpA**	**Helicase**	**50S**
**SrmB**	**Helicase**	**50S**
RhlE	Helicase	50S
DnaK/DnaJ/GrpE	Chaperone	30S, 50S
GroES/GroEL	Chaperone	50S
**Era**	**GTPase**	**30S**
RsgA	GTPase	30S
LepA	GTPase	30S
**Der**	**GTPase**	**50S**
YihA	GTPase	50S
**ObgE**	**GTPase**	**50S**
**BipA**	**GTPase**	**50S**

*Ten out of 24 proteins have previously been reported to play a role in cold sensitivity when deleted or mutated (bold).*

## Data Availability Statement

The raw data supporting the conclusions of this article will be made available by the authors, without undue reservation.

## Author Contributions

Y-GG directed the project. KJG performed most of the experiments. RE, J-EP, and BK participated in some of the experiments. KJG, X-FY, JZ, SKS, and Y-GG analyzed the data. KJG, RE, X-FY, and Y-GG wrote the manuscript with some input from all other co-authors.

## Conflict of Interest

The authors declare that the research was conducted in the absence of any commercial or financial relationships that could be construed as a potential conflict of interest.

## References

[B1] Awano N. Rajagopal V. Arbing M. Patel S. Hunt J. Inouye M. (2010). *Escherichia coli* RNase R has dual activities, helicase and RNase. *J. Bacteriol.* 192 1344–1352. 10.1128/jb.01368-09 20023028 PMC2820863

[B2] Awano N. Xu C. Ke H. Inoue K. Inouye M. Phadtare S. (2007). Complementation analysis of the cold-sensitive phenotype of the *Escherichia coli csdA* deletion strain. *J. Bacteriol.* 189 5808–5815. 10.1128/jb.00655-07 17557820 PMC1952031

[B3] Baba T. Ara T. Hasegawa M. Takai Y. Okumura Y. Baba M. (2006). Construction of *Escherichia coli* K-12 in-frame, single-gene knockout mutants: the Keio collection. *Mol. Syst. Biol.* 2:2006.0008. 10.1038/msb4100050 PMC1681482 16738554

[B4] Barria C. Malecki M. Arraiano C. (2013). Bacterial adaptation to cold. *Microbiology* 159 2437–2443. 10.1099/mic.0.052209-0 24068238

[B5] Bosl A. Böck A. (1981). Ribosomal mutation in *Escherichia coli* affecting membrane stability. *Mol. Gen. Genet.* 182 358–360. 10.1007/bf00269684 7026977

[B6] Cairrão F. Cruz A. Mori H. Arraiano C. M. (2003). Cold shock induction of RNase R and its role in the maturation of the quality control mediator SsrA/tmRNA. *Mol. Microbiol.* 50 1349–1360. 10.1046/j.1365-2958.2003.03766.x 14622421

[B7] Charollais J. Dreyfus M. Iost I. (2004). CsdA, a cold-shock RNA helicase from *Escherichia coli*, is involved in the biogenesis of 50S ribosomal subunit. *Nucleic Acids Res.* 32 2751–2759. 10.1093/nar/gkh603 15148362 PMC419605

[B8] Charollais J. Pflieger D. Vinh J. Dreyfus M. Iost I. (2003). The DEAD-box RNA helicase SrmB is involved in the assembly of 50S ribosomal subunits in *Escherichia coli*. *Mol. Microbiol.* 48 1253–1265. 10.1046/j.1365-2958.2003.03513.x 12787353

[B9] Choi E. Hwang J. (2018). The GTPase BipA expressed at low temperature in *Escherichia coli* assists ribosome assembly and has chaperone-like activity. *J. Biol. Chem.* 293 18404–18419. 10.1074/jbc.ra118.002295 30305394 PMC6254333

[B10] Choudhury P. Flower A. M. (2015). Efficient assembly of ribosomes is inhibited by deletion of *bipA* in *Escherichia coli*. *J. Bacteriol.* 197 1819–1827. 10.1128/jb.00023-15 25777676 PMC4402399

[B11] Conrad J. Sun D. Englund N. Ofengand J. (1998). The *rluC* gene of *Escherichia coli* codes for a pseudouridine synthase that is solely responsible for synthesis of pseudouridine at positions 955, 2504, and 2580 in 23 S ribosomal RNA. *J. Biol. Chem.* 273 18562–18566. 10.1074/jbc.273.29.18562 9660827

[B12] Cremer J. Honda T. Tang Y. Wong-Ng J. Vergassola M. Hwa T. (2019). Chemotaxis as a navigation strategy to boost range expansion. *Nature* 575 658–663. 10.1038/s41586-019-1733-y 31695195 PMC6883170

[B13] Davis J. H. Tan Y. Z. Carragher B. Potter C. S. Lyumkis D. Williamson J. R. (2016). Modular assembly of the bacterial large ribosomal subunit. *Cell* 167 1610–1622.e5. 27912064 10.1016/j.cell.2016.11.020 PMC5145266

[B14] Del Peso Santos T. Alvarez L. Sit B. Irazoki O. Blake J. Warner B. R. (2021). BipA exerts temperature-dependent translational control of biofilm-associated colony morphology in *Vibrio cholerae*. *Elife* 10:e60607. 10.7554/eLife.60607 PMC7886329 33588990

[B15] deLivron M. A. Makanji H. S. Lane M. C. Robinson V. L. (2009). A novel domain in translational GTPase BipA mediates interaction with the 70S ribosome and influences GTP hydrolysis. *Biochemistry* 48 10533–10541. 10.1021/bi901026z 19803466

[B16] Ero R. Kumar V. Chen Y. Gao Y. G. (2016). Similarity and diversity of translational GTPase factors EF-G, EF4, and BipA: From structure to function. *RNA Biol.* 13 1258–1273. 10.1080/15476286.2016.1201627 27325008 PMC5207388

[B17] Fan H. Hahm J. Diggs S. Perry J. J. P. Blaha G. (2015). Structural and functional analysis of BipA, a regulator of virulence in enteropathogenic *Escherichia coli*. *J. Biol. Chem.* 290 20856–20864. 10.1074/jbc.m115.659136 26163516 PMC4543647

[B18] Gao Y.-G. Selmer M. Dunham C. M. Weixlbaumer A. Kelley A. C. Ramakrishnan V. (2009). The structure of the ribosome with elongation factor G trapped in the posttranslocational state. *Science* 326 694–699. 10.1126/science.1179709 19833919 PMC3763468

[B19] Gibbs M. R. Fredrick K. (2018). Roles of elusive translational GTPases come to light and inform on the process of ribosome biogenesis in bacteria. *Mol. Microbiol.* 107 445–454. 10.1111/mmi.13895 29235176 PMC5796857

[B20] Gibbs M. R. Moon K. M. Warner B. R. Chen M. Bundschuh R. Foster L. J. (2020). Functional analysis of BipA in *E. coli* reveals the natural plasticity of 50s subunit assembly. *J. Mol. Biol.* 432 5259–5272. 10.1016/j.jmb.2020.07.013 32710983 PMC7502552

[B21] Grant A. J. Farris M. Alefounder P. Williams P. H. Woodward M. J. O’Connor C. D. (2003). Co-ordination of pathogenicity island expression by the BipA GTPase in enteropathogenic *Escherichia coli* (EPEC). *Mol. Microbiol.* 48 507–521. 10.1046/j.1365-2958.2003.t01-1-03447.x 12675808

[B22] Guan Y.-X. Pan H.-X. Gao Y.-G. Yao S.-J. Cho M.-G. (2005). Refolding and purification of recombinant human interferon-γ expressed as inclusion bodies in *Escherichia coli* using size exclusion chromatography. *Biotechnol. Bioprocess Eng.* 10 122–127. 10.1007/bf02932581

[B23] Hauryliuk V. Atkinson G. C. Murakami K. S. Tenson T. Gerdes K. (2015). Recent functional insights into the role of (p) ppGpp in bacterial physiology. *Nat. Rev. Microbiol.* 13 298–309. 10.1038/nrmicro3448 25853779 PMC4659695

[B24] Heermann R. Zeppenfeld T. Jung K. (2008). Simple generation of site-directed point mutations in the *Escherichia coli* chromosome using Red^®^/ET^®^ Recombination. *Microb. Cell Factor.* 7:14. 10.1186/1475-2859-7-14 18435843 PMC2373285

[B25] Jain C. (2008). The *E. coli* RhlE RNA helicase regulates the function of related RNA helicases during ribosome assembly. *RNA* 14 381–389. 10.1261/rna.800308 18083833 PMC2212244

[B26] Jiang M. Datta K. Walker A. Strahler J. Bagamasbad P. Andrews P. C. (2006). The *Escherichia coli* GTPase CgtAE is involved in late steps of large ribosome assembly. *J. Bacteriol.* 188 6757–6770. 10.1128/jb.00444-06 16980477 PMC1595513

[B27] Kalogeraki V. S. Winans S. C. (1997). Suicide plasmids containing promoterless reporter genes can simultaneously disrupt and create fusions to target genes of diverse bacteria. *Gene* 188 69–75. 10.1016/s0378-1119(96)00778-0 9099861

[B28] Kierzek E. Malgowska M. Lisowiec J. Turner D. H. Gdaniec Z. Kierzek R. (2013). The contribution of pseudouridine to stabilities and structure of RNAs. *Nucleic acids Res.* 42 3492–3501. 10.1093/nar/gkt1330 24369424 PMC3950712

[B29] Kinosita Y. Ishida T. Yoshida M. Ito R. Morimoto Y. V. Goto K. (2020). Distinct chemotactic behavior in the original *Escherichia coli* K-12 depending on forward-and-backward swimming, not on run-tumble movements. *Sci. Rep.* 10:15887. 10.1038/s41598-020-72429-1 PMC7522084 32985511

[B30] Kitahara K. Suzuki T. (2009). The ordered transcription of RNA domains is not essential for ribosome biogenesis in *Escherichia coli*. *Mol. Cell* 34 760–766. 10.1016/j.molcel.2009.05.014 19560426

[B31] Koripella R. K. Holm M. Dourado D. Mandava C. S. Flores S. Sanyal S. (2015). A conserved histidine in switch-II of EF-G moderates release of inorganic phosphate. *Sci. Rep.* 5:12970. 10.1038/srep12970 PMC4532990 26264741

[B32] Koster D. A. Mayo A. Bren A. Alon U. (2012). Surface growth of a motile bacterial population resembles growth in a chemostat. *J. Mol. Biol.* 424 180–191. 10.1016/j.jmb.2012.09.005 23000812

[B33] Krishnan K. Flower A. M. (2008). Suppression of Δ*bipA* phenotypes in *Escherichia coli* by abolishment of pseudouridylation at specific sites on the 23S rRNA. *J. Bacteriol.* 190 7675–7683. 10.1128/jb.00835-08 18820021 PMC2583627

[B34] Kumar V. Chen Y. Ero R. Ahmed T. Tan J. Li Z. (2015). Structure of BipA in GTP form bound to the ratcheted ribosome. *Proc. Natl. Acad. Sci. U.S.A.* 112 10944–10949. 10.1073/pnas.1513216112 26283392 PMC4568239

[B35] Kumar V. Ero R. Ahmed T. Goh K. J. Zhan Y. Bhushan S. (2016). Structure of the GTP Form of Elongation Factor 4 (EF4) Bound to the Ribosome. *J. Biol. Chem.* 291 12943–12950. 10.1074/jbc.m116.725945 27137929 PMC4933213

[B36] Liu W. Cremer J. Li D. Hwa T. Liu C. (2019). An evolutionarily stable strategy to colonize spatially extended habitats. *Nature* 575 664–668. 10.1038/s41586-019-1734-x 31695198 PMC6883132

[B37] Martínez-García E. Nikel P. I. Chavarría M. de Lorenzo V. (2014). The metabolic cost of flagellar motion in *Pseudomonas putida* KT 2440. *Environ. Microbiol.* 16 291–303. 10.1111/1462-2920.12309 24148021

[B38] Murakami K. Ono T. Viducic D. Kayama S. Mori M. Hirota K. (2005). Role for *rpoS* gene of *Pseudomonas aeruginosa* in antibiotic tolerance. *FEMS Microbiol. Lett.* 242 161–167. 15621433 10.1016/j.femsle.2004.11.005

[B39] Neidig A. Yeung A. T. Rosay T. Tettmann B. Strempel N. Rueger M. (2013). TypA is involved in virulence, antimicrobial resistance and biofilm formation in *Pseudomonas aeruginosa*. *BMC Microbiol.* 13:77. 10.1186/1471-2180-13-77 23570569 PMC3639842

[B40] Ottemann K. M. Miller J. F. (1997). Roles for motility in bacterial–host interactions. *Mol. Microbiol.* 24 1109–1117. 10.1046/j.1365-2958.1997.4281787.x 9218761

[B41] Park J. E. Dutta B. Tse S. W. Gupta N. Tan C. F. Low J. K. (2019). Hypoxia-induced tumor exosomes promote M2-like macrophage polarization of infiltrating myeloid cells and microRNA-mediated metabolic shift. *Oncogene* 38 5158–5173. 10.1038/s41388-019-0782-x 30872795

[B42] Pech M. Karim Z. Yamamoto H. Kitakawa M. Qin Y. Nierhaus K. H. (2011). Elongation factor 4 (EF4/LepA) accelerates protein synthesis at increased Mg2+ concentrations. *Proc. Natl. Acad. Sci. U.S.A.* 108 3199–3203. 10.1073/pnas.1012994108 21300907 PMC3044372

[B43] Peil L. Virumäe K. Remme J. (2008). Ribosome assembly in *Escherichia coli* strains lacking the RNA helicase DeaD/CsdA or DbpA. *FEBS J.* 275 3772–3782. 10.1111/j.1742-4658.2008.06523.x 18565105

[B44] Pfennig P. Flower A. (2001). BipA is required for growth of *Escherichia coli* K12 at low temperature. *Mol. Genet. Genom.* 266 313–317. 10.1007/s004380100559 11683274

[B45] Phadtare S. (2011). Unwinding activity of cold shock proteins and RNA metabolism. *RNA Biol.* 8 394–397. 10.4161/rna.8.3.14823 21445001 PMC3218510

[B46] Phadtare S. Hwang J. Severinov K. Inouye M. (2003). CspB and CspL, thermostable cold-shock proteins from *Thermotoga maritima*. *Genes Cells* 8:801. 10.1046/j.1365-2443.2003.00675.x 14531859

[B47] Philippe N. Alcaraz J.-P. Coursange E. Geiselmann J. Schneider D. (2004). Improvement of pCVD442, a suicide plasmid for gene allele exchange in bacteria. *Plasmid* 51 246–255. 10.1016/j.plasmid.2004.02.003 15109831

[B48] Prud’homme-Généreux A. Beran R. K. Iost I. Ramey C. S. Mackie G. A. Simons R. W. (2004). Physical and functional interactions among RNase E, polynucleotide phosphorylase and the cold-shock protein, CsdA: evidence for a ‘cold shock degradosome’. *Mol. Microbiol.* 54 1409–1421. 10.1111/j.1365-2958.2004.04360.x 15554978

[B49] Qi S. Y. Li Y. Szyroki A. Giles I. G. Moir A. David O’Connor C. (1995). *Salmonella typhimurium* responses to a bactericidal protein from human neutrophils. *Mol. Microbiol.* 17 523–531. 10.1111/j.1365-2958.1995.mmi_17030523.x 8559071

[B50] Resch A. Većerek B. Palavra K. Bläsi U. (2010). Requirement of the CsdA DEAD-box helicase for low temperature riboregulation of *rpoS* mRNA. *RNA Biol.* 7 796–802. 10.4161/rna.7.6.13768 21045550 PMC3073337

[B51] Rudra D. Warner J. R. (2004). What better measure than ribosome synthesis? *Genes Dev.* 18 2431–2436. 10.1101/gad.1256704 15489289

[B52] Sato A. Kobayashi G. Hayashi H. Yoshida H. Wada A. Maeda M. (2005). The GTP binding protein Obg homolog ObgE is involved in ribosome maturation. *Genes Cells* 10 393–408. 10.1111/j.1365-2443.2005.00851.x 15836769

[B53] Scarano G. I Krab M. Bocchini V. Parmeggiani A. (1995). Relevance of histidine-84 in the elongation factor Tu GTPase activity and in poly (Phe) synthesis: its substitution by glutamine and alanine. *FEBS Lett.* 365 214–218. 10.1016/0014-5793(95)00469-p 7781781

[B54] Schaefer J. Jovanovic G. Kotta-Loizou I. Buck M. (2016). Single-step method for β-galactosidase assays in *Escherichia coli* using a 96-well microplate reader. *Anal. Biochem.* 503 56–57. 10.1016/j.ab.2016.03.017 27036618 PMC4865525

[B55] Schmeing T. M. Voorhees R. M. Kelley A. C. Gao Y. G. Murphy F. V. IV Weir J. R. (2009). The crystal structure of the ribosome bound to EF-Tu and aminoacyl-tRNA. *Science* 326 688–694. 19833920 10.1126/science.1179700 PMC3763470

[B56] Selmer M. Gao Y. G. Weixlbaumer A. Ramakrishnan V. (2012). Ribosome engineering to promote new crystal forms. *Acta Crystallogr. D Biol. Crystallogr.* 68(Pt 5) 578–583. 10.1107/s0907444912006348 22525755 PMC3335287

[B57] Shigeno Y. Uchiumi T. Nomura T. (2016). Involvement of ribosomal protein L6 in assembly of functional 50S ribosomal subunit in *Escherichia coli* cells. *Biochem. Biophys. Res. Commun.* 473 237–242. 10.1016/j.bbrc.2016.03.085 27003253

[B58] Tanaka Y. Sakamoto S. Kuroda M. Goda S. Gao Y. G. Tsumoto K. (2008). A helical string of alternately connected three-helix bundles for the cell wall-associated adhesion protein Ebh from *Staphylococcus aureus*. *Structure* 16 488–496. 10.1016/j.str.2007.12.018 18334223

[B59] Thompson A. Schäfer J. Kuhn K. Kienle S. Schwarz J. Schmidt G. (2003). Tandem mass tags: a novel quantification strategy for comparative analysis of complex protein mixtures by MS/MS. *Anal. Chem.* 75 1895–1904. 10.1021/ac0262560 12713048

[B60] Toh S.-M. Mankin A. S. (2008). An indigenous posttranscriptional modification in the ribosomal peptidyl transferase center confers resistance to an array of protein synthesis inhibitors. *J. Mol. Biol.* 380 593–597. 10.1016/j.jmb.2008.05.027 18554609 PMC5367387

[B61] Uppal S. Jawali N. (2015). Cyclic AMP receptor protein (CRP) regulates the expression of *cspA, cspB, cspG* and *cspI*, members of *cspA* family, in *Escherichia coli*. *Arch. Microbiol.* 197 497–501. 10.1007/s00203-015-1085-4 25637299

[B62] Vesper O. Amitai S. Belitsky M. Byrgazov K. Kaberdina A. C. Engelberg-Kulka H. (2011). Selective translation of leaderless mRNAs by specialized ribosomes generated by MazF in *Escherichia coli*. *Cell* 147 147–157. 10.1016/j.cell.2011.07.047 21944167 PMC4894548

[B63] Yang C. Cui C. Ye Q. Kan J. Fu S. Song S. (2017). *Burkholderia cenocepacia* integrates cis-2-dodecenoic acid and cyclic dimeric guanosine monophosphate signals to control virulence. *Proc. Natl. Acad. Sci. U.S.A.* 114 13006–13011. 10.1073/pnas.1709048114 29158389 PMC5724260

[B64] Yu Y. Wu Y. Cao B. Gao Y.-G. Yan X. (2015). Adjustable bidirectional extracellular electron transfer between *Comamonas testosteroni* biofilms and electrode via distinct electron mediators. *Electrochem. Commun.* 59 43–47. 10.1016/j.elecom.2015.07.007

[B65] Zheng J. Li N. Tan Y. P. Sivaraman J. Mok Y.-K. Mo Z. L. (2007). EscC is a chaperone for the *Edwardsiella tarda* type III secretion system putative translocon components EseB and EseD. *Microbiology* 153 1953–1962. 10.1099/mic.0.2006/004952-0 17526852

