# Supplementary material for: Translational GTPase BipA Is Involved in the Maturation of a Large Subunit of Bacterial Ribosome at Suboptimal Temperature
Source: Front Microbiol. 2021 Jul 13;12:686049. doi: 10.3389/fmicb.2021.686049 (PMC8313970; doi:10.3389/fmicb.2021.686049)

# ­Translational GTPase BipA is involved in the maturation of large subunit of bacterial ribosome at suboptimal temperature

10.3389/fmicb.2021.686049

# Supporting information

**Supplementary movie 1** Microscopy analysis of bacteria swimming motility of K12WT. Overnight culture was diluted to OD_600_ = 0.1, and 100 μL of diluted culture per well.

**Supplementary movie 2** Microscopy analysis of bacteria swimming motility of Δ*bipA*. Overnight culture was diluted to OD_600_ = 0.1, and 100 μL of diluted culture per well.

**Supplementary movie 3** Microscopy analysis of bacteria swimming motility of Δ*bipA* (pCA24N-BipA). Overnight culture was diluted to OD_600_ = 0.1, and 100 μL of diluted culture per well.

**Supplementary movie 4** Microscopy analysis of bacteria swimming motility of Δ*rluC* Δ*bipA*. Overnight culture was diluted to OD_600_ = 0.1, and 100 μL of diluted culture per well.

**Table S5** Differential protein expression determined by TMT-MS with FC of at least 1.5 X between Δ*bipA* and K12WT under cold shock.

| Gene | FC* | Gene product |
| --- | --- | --- |
| typA | -3.36 | GTP-binding protein TypA/BipA |
| fliN | -1.86 | Flagellar motor switch protein FliN |
| ilvC | -1.74 | Ketol-acid reductoisomerase (NADP(+)) |
| proW | -1.57 | Glycine betaine/proline betaine transport system permease protein ProW |
| rplM | 1.50 | 50S ribosomal protein L13 |
| astC | 1.51 | Succinylornithine transaminase |
| lldP | 1.52 | L-lactate permease |
| actP | 1.52 | Cation/acetate symporter ActP |
| rnr | 1.53 | Ribonuclease R |
| mdtK | 1.53 | Multidrug resistance protein MdtK |
| rluB | 1.53 | Ribosomal large subunit pseudouridine synthase B |
| yadG | 1.55 | Uncharacterized ABC transporter ATP-binding protein YadG |
| degP | 1.57 | Periplasmic serine endoprotease DegP |
| fadB | 1.58 | Fatty acid oxidation complex subunit alpha |
| raiA | 1.58 | Ribosome-associated inhibitor A |
| eptB | 1.59 | Kdo(2)-lipid A phosphoethanolamine 7''-transferase |
| rpoH | 1.60 | RNA polymerase sigma factor RpoH |
| acs | 1.64 | Acetyl-coenzyme A synthetase |
| ygiS | 1.70 | Probable deoxycholate-binding periplasmic protein YgiS |
| idi | 1.72 | Isopentenyl-diphosphate Delta-isomerase |
| nrdF | 1.73 | Ribonucleoside-diphosphate reductase 2 subunit beta |
| mtlA | 1.74 | PTS system mannitol-specific EIICBA component |
| cspA | 1.75 | Cold shock protein CspA |
| rpoS | 1.75 | RNA polymerase sigma factor RpoS |
| deaD | 1.76 | ATP-dependent RNA helicase DeaD |
| obgE | 1.78 | GTPase ObgE/CgtA |
| yhcN | 1.80 | Uncharacterized protein YhcN |
| mhpR | 2.05 | DNA-binding transcriptional activator MhpR |
| puuB | 2.09 | Gamma-glutamylputrescine oxidoreductase |

*** FC, fold-change, positive values refer to upregulation, negative values refer to downregulation.**

**Table S6** Differential protein expression determined by TMT-MS with FC of at least 1.5 X between Δ*bipA* (pCA24N-BipA) and K12WT under cold shock.

| Gene | FC* | Product |
| --- | --- | --- |
| yjbJ | -2.17 | UPF0337 protein YjbJ |
| gadA | -1.87 | Glutamate decarboxylase alpha |
| yodD | -1.85 | Uncharacterized protein YodD |
| mdtE | -1.85 | Multidrug resistance protein MdtE |
| hyaB | -1.84 | Hydrogenase-1 large chain |
| yhfZ | -1.71 | Uncharacterized protein YhfZ |
| elaB | -1.68 | Protein ElaB |
| proW | -1.66 | Glycine betaine/proline betaine transport system permease protein ProW |
| yhbO | -1.65 | Protein/nucleic acid deglycase 2 |
| osmY | -1.60 | Osmotically-inducible protein Y |
| slp | -1.57 | Outer membrane protein Slp |
| blc | -1.52 | Outer membrane lipoprotein Blc |
| flhE | 1.51 | Flagellar protein FlhE |
| modA | 1.56 | Molybdate-binding protein ModA |
| fliY | 1.56 | L-cystine-binding protein FliY |
| flgC | 1.56 | Flagellar basal-body rod protein FlgC |
| ilvB | 1.57 | Acetolactate synthase isozyme 1 large subunit |
| pptA | 1.57 | Tautomerase PptA |
| ptsG | 1.59 | PTS system glucose-specific EIICB component |
| cutC | 1.65 | Copper homeostasis protein CutC |
| ilvN | 1.69 | Acetolactate synthase isozyme 1 small subunit |
| fliH | 1.72 | Flagellar assembly protein FliH |
| fliM | 1.73 | Flagellar motor switch protein FliM |
| fliI | 1.89 | Flagellum-specific ATP synthase |
| fliO | 1.89 | Flagellar protein FliO |
| fliA | 1.90 | RNA polymerase sigma factor FliA |
| ompA | 1.99 | Outer membrane protein A |
| flgI | 2.00 | Flagellar P-ring protein |
| ibpB | 2.02 | Small heat shock protein IbpB |
| flhA | 2.10 | Flagellar biosynthesis protein FlhA |
| ompF | 2.20 | Outer membrane protein F |
| yhjX | 2.25 | Uncharacterized MFS-type transporter YhjX |
| fliP | 2.26 | Flagellar biosynthetic protein FliP |
| ompX | 2.31 | Outer membrane protein X |
| fliF | 2.33 | Flagellar M-ring protein |
| sdaC | 2.39 | Serine transporter |
| flgH | 2.47 | Flagellar L-ring protein |
| fliG | 2.48 | Flagellar motor switch protein FliG |
| nepI | 2.56 | Purine ribonucleoside efflux pump NepI |
| flgK | 2.62 | Flagellar hook-associated protein 1 |
| flgA | 2.66 | Flagella basal body P-ring formation protein FlgA |
| aer | 2.66 | Aerotaxis receptor |
| fliS | 2.72 | Flagellar secretion chaperone FliS |
| flgD | 2.81 | Basal-body rod modification protein FlgD |
| fliD | 2.88 | Flagellar hook-associated protein 2 |
| flgE | 2.89 | Flagellar hook protein FlgE |
| cheA | 2.90 | Chemotaxis protein CheA |
| fliT | 3.04 | Flagellar protein FliT |
| flgL | 3.12 | Flagellar hook-associated protein 3 |
| pdeH | 3.17 | Cyclic di-GMP phosphodiesterase PdeH |
| flgG | 3.22 | Flagellar basal-body rod protein FlgG |
| fliZ | 3.23 | Regulator of sigma S factor FliZ |
| fliN | 3.53 | Flagellar motor switch protein FliN |
| cheZ | 3.63 | Protein phosphatase CheZ |
| tar | 3.69 | Methyl-accepting chemotaxis protein II |
| motA | 3.71 | Motility protein A |
| fliL | 3.86 | Flagellar protein FliL |
| ycgR | 3.97 | Flagellar brake protein YcgR |
| trg | 4.00 | Methyl-accepting chemotaxis protein III |
| cheB | 4.16 | Protein-glutamate methylesterase/protein-glutamine glutaminase |
| flgN | 4.26 | Flagella synthesis protein FlgN |
| tsr | 4.38 | Methyl-accepting chemotaxis protein I |
| motB | 4.54 | Motility protein B |
| cheA" | 4.76 | Isoform cheA(S) of Chemotaxis protein CheA |
| cheR | 4.77 | Chemotaxis protein methyltransferase |
| tap | 5.06 | Methyl-accepting chemotaxis protein IV |
| cheY | 5.15 | Chemotaxis protein CheY |
| lacI | 5.57 | Lactose operon repressor |
| fliC | 6.33 | Flagellin |
| cheW | 6.90 | Chemotaxis protein CheW |

*** FC, fold-change, positive values refer to upregulation, negative values refer to downregulation.**

**Table S7** Differential protein expression determined by TMT-MS with FC of at least 1.5 X between Δ*bipA* (pCA24N-BipA) and K12WT under cold shock.

| Gene | FC* | Product |
| --- | --- | --- |
| ytfK | -2.93 | Uncharacterized protein YtfK |
| yjbJ | -2.50 | UPF0337 protein YjbJ |
| rpoS | -2.30 | RNA polymerase sigma factor RpoS |
| hdeA | -2.19 | Acid stress chaperone HdeA |
| mhpR | -2.15 | DNA-binding transcriptional activator MhpR |
| yodD | -2.04 | Uncharacterized protein YodD |
| puuB | -1.96 | Gamma-glutamylputrescine oxidoreductase |
| gadA | -1.89 | Glutamate decarboxylase alpha |
| osmY | -1.88 | Osmotically-inducible protein Y |
| elaB | -1.86 | Protein ElaB |
| raiA | -1.86 | Ribosome-associated inhibitor A |
| deaD | -1.86 | ATP-dependent RNA helicase DeaD |
| yhbO | -1.84 | Protein/nucleic acid deglycase 2 |
| adhP | -1.83 | Alcohol dehydrogenase, propanol-preferring |
| idi | -1.81 | Isopentenyl-diphosphate Delta-isomerase |
| cspA | -1.80 | Cold shock protein CspA |
| degP | -1.80 | Periplasmic serine endoprotease DegP |
| hdeB | -1.78 | Acid stress chaperone HdeB |
| blc | -1.77 | Outer membrane lipoprotein Blc |
| obgE | -1.76 | GTPase ObgE/CgtA |
| ygiS | -1.74 | Probable deoxycholate-binding periplasmic protein YgiS |
| hchA | -1.74 | Protein/nucleic acid deglycase 1 |
| talA | -1.73 | Transaldolase A |
| ygaM | -1.73 | Uncharacterized protein YgaM |
| ygiW | -1.71 | Protein YgiW |
| yghA | -1.70 | Uncharacterized oxidoreductase YghA |
| rpoH | -1.69 | RNA polymerase sigma factor RpoH |
| nrdF | -1.68 | Ribonucleoside-diphosphate reductase 2 subunit beta |
| mdtE | -1.68 | Multidrug resistance protein MdtE |
| fadB | -1.68 | Fatty acid oxidation complex subunit alpha |
| yqjD | -1.67 | Uncharacterized protein YqjD |
| yegP | -1.67 | UPF0339 protein YegP |
| rnr | -1.63 | Ribonuclease R |
| caiA | -1.62 | Crotonobetainyl-CoA reductase |
| katE | -1.61 | Catalase HPII |
| hcaR | -1.60 | Hca operon transcriptional activator HcaR |
| nfsA | -1.59 | Oxygen-insensitive NADPH nitroreductase |
| rluB | -1.58 | Ribosomal large subunit pseudouridine synthase B |
| upp | -1.58 | Uracil phosphoribosyltransferase |
| aceB | -1.55 | Malate synthase A |
| poxB | -1.54 | Pyruvate dehydrogenase [ubiquinone] |
| astC | -1.52 | Succinylornithine transaminase |
| ygiM | -1.52 | Uncharacterized protein YgiM |
| slp | -1.52 | Outer membrane protein Slp |
| garR | -1.52 | 2-hydroxy-3-oxopropionate reductase |
| eco | -1.51 | Ecotin |
| rplM | -1.51 | 50S ribosomal protein L13 |
| fecB | 1.51 | Fe(3+) dicitrate-binding periplasmic protein |
| glpT | 1.52 | Glycerol-3-phosphate transporter |
| cutC | 1.55 | Copper homeostasis protein CutC |
| glpA | 1.55 | Anaerobic glycerol-3-phosphate dehydrogenase subunit A |
| modA | 1.56 | Molybdate-binding protein ModA |
| glpC | 1.57 | Anaerobic glycerol-3-phosphate dehydrogenase subunit C |
| ompC | 1.63 | Outer membrane protein C |
| flhE | 1.67 | Flagellar protein FlhE |
| thrB | 1.72 | Homoserine kinase |
| sdaC | 1.73 | Serine transporter |
| fliH | 1.75 | Flagellar assembly protein FliH |
| fliI | 1.92 | Flagellum-specific ATP synthase |
| ilvN | 1.92 | Acetolactate synthase isozyme 1 small subunit |
| fliM | 1.93 | Flagellar motor switch protein FliM |
| ilvB | 1.94 | Acetolactate synthase isozyme 1 large subunit |
| ompA | 1.94 | Outer membrane protein A |
| fliA | 1.98 | RNA polymerase sigma factor FliA |
| flhA | 1.99 | Flagellar biosynthesis protein FlhA |
| fliO | 2.00 | Flagellar protein FliO |
| fecA | 2.01 | Fe(3+) dicitrate transport protein FecA |
| nepI | 2.11 | Purine ribonucleoside efflux pump NepI |
| flgI | 2.19 | Flagellar P-ring protein |
| ompX | 2.25 | Outer membrane protein X |
| ompF | 2.32 | Outer membrane protein F |
| yhjX | 2.37 | Uncharacterized MFS-type transporter YhjX |
| ilvC | 2.40 | Ketol-acid reductoisomerase (NADP(+)) |
| fliP | 2.71 | Flagellar biosynthetic protein FliP |
| fliF | 2.73 | Flagellar M-ring protein |
| fliD | 2.80 | Flagellar hook-associated protein 2 |
| flgE | 2.81 | Flagellar hook protein FlgE |
| fliS | 2.84 | Flagellar secretion chaperone FliS |
| fliG | 2.85 | Flagellar motor switch protein FliG |
| flgK | 2.87 | Flagellar hook-associated protein 1 |
| flgA | 2.94 | Flagella basal body P-ring formation protein FlgA |
| cheA | 3.01 | Chemotaxis protein CheA |
| flgH | 3.02 | Flagellar L-ring protein |
| aer | 3.03 | Aerotaxis receptor |
| typA | 3.05 | GTP-binding protein TypA/BipA |
| fliT | 3.14 | Flagellar protein FliT |
| flgG | 3.20 | Flagellar basal-body rod protein FlgG |
| pdeH | 3.26 | Cyclic di-GMP phosphodiesterase PdeH |
| cheZ | 3.40 | Protein phosphatase CheZ |
| flgL | 3.60 | Flagellar hook-associated protein 3 |
| flgD | 3.73 | Basal-body rod modification protein FlgD |
| fliZ | 3.83 | Regulator of sigma S factor FliZ |
| motA | 3.84 | Motility protein A |
| ycgR | 3.94 | Flagellar brake protein YcgR |
| trg | 4.22 | Methyl-accepting chemotaxis protein III |
| tsr | 4.31 | Methyl-accepting chemotaxis protein I |
| cheB | 4.37 | Protein-glutamate methylesterase/protein-glutamine glutaminase |
| tar | 4.44 | Methyl-accepting chemotaxis protein II |
| cheR | 4.62 | Chemotaxis protein methyltransferase |
| flgN | 4.69 | Flagella synthesis protein FlgN |
| motB | 4.71 | Motility protein B |
| cheA" | 4.82 | Isoform cheA(S) of Chemotaxis protein CheA |
| fliL | 5.17 | Flagellar protein FliL |
| tap | 5.21 | Methyl-accepting chemotaxis protein IV |
| cheY | 5.27 | Chemotaxis protein CheY |
| lacI | 5.30 | Lactose operon repressor |
| fliC | 6.30 | Flagellin |
| fliN | 6.56 | Flagellar motor switch protein FliN |
| cheW | 7.35 | Chemotaxis protein CheW |

*** FC, fold-change, positive values refer to upregulation, negative values refer to downregulation.**

**Table S8** Differential protein expression determined by TMT-MS with FC of at least 1.5 X between Δ*rluC* and K12WT under cold shock.

| Gene | FC* | Product |
| --- | --- | --- |
| rluC | -2.43 | Ribosomal large subunit pseudouridine synthase C |
| rpoS | 1.52 | RNA polymerase sigma factor RpoS |
| blc | 1.56 | Outer membrane lipoprotein Blc |
| gatD | 1.58 | Galactitol 1-phosphate 5-dehydrogenase |
| narH | 1.80 | Respiratory nitrate reductase 1 beta chain |
| narG | 1.92 | Respiratory nitrate reductase 1 alpha chain |
| grcA | 2.29 | Autonomous glycyl radical cofactor |

*** FC, fold-change, positive values refer to upregulation, negative values refer to downregulation.**

**Table S9** Differential protein expression determined by TMT-MS with FC of at least 1.5 X between Δ*rluC/*Δ*bipA* and K12WT under cold shock.

| Gene | FC* | Product |
| --- | --- | --- |
| typA | -3.18 | GTP-binding protein TypA/BipA |
| rluC | -2.17 | Ribosomal large subunit pseudouridine synthase C |
| yhfZ | -1.70 | Uncharacterized protein YhfZ |
| nei | -1.60 | Endonuclease 8 |
| ppnN | -1.55 | Pyrimidine/purine nucleotide 5'-monophosphate nucleosidase |
| mgtA | 1.52 | Magnesium-transporting ATPase, P-type 1 |
| fadB | 1.52 | Fatty acid oxidation complex subunit alpha |
| yadG | 1.55 | Uncharacterized ABC transporter ATP-binding protein YadG |
| lldP | 1.55 | L-lactate permease |
| raiA | 1.56 | Ribosome-associated inhibitor A |
| cspD | 1.56 | Cold shock-like protein CspD |
| malM | 1.58 | Maltose operon periplasmic protein |
| nanE | 1.58 | Putative N-acetylmannosamine-6-phosphate 2-epimerase |
| gatD | 1.58 | Galactitol 1-phosphate 5-dehydrogenase |
| tdcE | 1.60 | PFL-like enzyme TdcE |
| uxaC | 1.60 | Uronate isomerase |
| yjhC | 1.61 | Uncharacterized oxidoreductase YjhC |
| astC | 1.61 | Succinylornithine transaminase |
| aldA | 1.63 | Lactaldehyde dehydrogenase |
| yibT | 1.64 | Uncharacterized protein YibT |
| aphA | 1.64 | Class B acid phosphatase |
| actP | 1.66 | Cation/acetate symporter ActP |
| fryB | 1.66 | PTS system fructose-like EIIB component 1 |
| nanA | 1.69 | N-acetylneuraminate lyase |
| malF | 1.69 | Maltose/maltodextrin transport system permease protein MalF |
| puuB | 1.78 | Gamma-glutamylputrescine oxidoreductase |
| acs | 1.79 | Acetyl-coenzyme A synthetase |
| narH | 1.80 | Respiratory nitrate reductase 1 beta chain |
| mhpR | 1.80 | DNA-binding transcriptional activator MhpR |
| lamB | 1.99 | Maltoporin |
| rpoS | 2.06 | RNA polymerase sigma factor RpoS |
| nanM | 2.30 | N-acetylneuraminate epimerase |

*** FC, fold-change, positive values refer to upregulation, negative values refer to downregulation.**

**Table S10** Differential protein expression determined by TMT-MS with FC of at least 1.5 X between Δ*rluC/*Δ*bipA* and Δ*bipA* under cold shock.

| Gene | FC* | Product |
| --- | --- | --- |
| rluC | -2.43 | Ribosomal large subunit pseudouridine synthase C |
| nrdF | -1.69 | Ribonucleoside-diphosphate reductase 2 subunit beta |
| obgE | -1.57 | GTPase ObgE/CgtA |
| deaD | -1.55 | ATP-dependent RNA helicase DeaD |
| cspA | -1.51 | Cold shock protein CspA |
| proW | 1.52 | Glycine betaine/proline betaine transport system permease protein ProW |
| pdxH | 1.55 | Pyridoxine/pyridoxamine 5'-phosphate oxidase |
| narG | 1.56 | Respiratory nitrate reductase 1 alpha chain |
| nirB | 1.63 | Nitrite reductase (NADH) large subunit |
| tdcE | 1.79 | PFL-like enzyme TdcE |
| fliN | 1.92 | Flagellar motor switch protein FliN |
| narH | 1.94 | Respiratory nitrate reductase 1 beta chain |
| grcA | 2.13 | Autonomous glycyl radical cofactor |

*** FC, fold-change, positive values refer to upregulation, negative values refer to downregulation.**

**Supplementary Figure S11** Close-up view on *E. coli* 70S ribosome interacting with BipA C-terminal loop (CTL). The A-loop was coloured in green and the nucleotides that formed the A-loop were labelled according from U2552 to C2556. The CTL of BipA was coloured in purple and could be seen projecting into the A-loop region of 23S rRNA to establish extensive contacts.


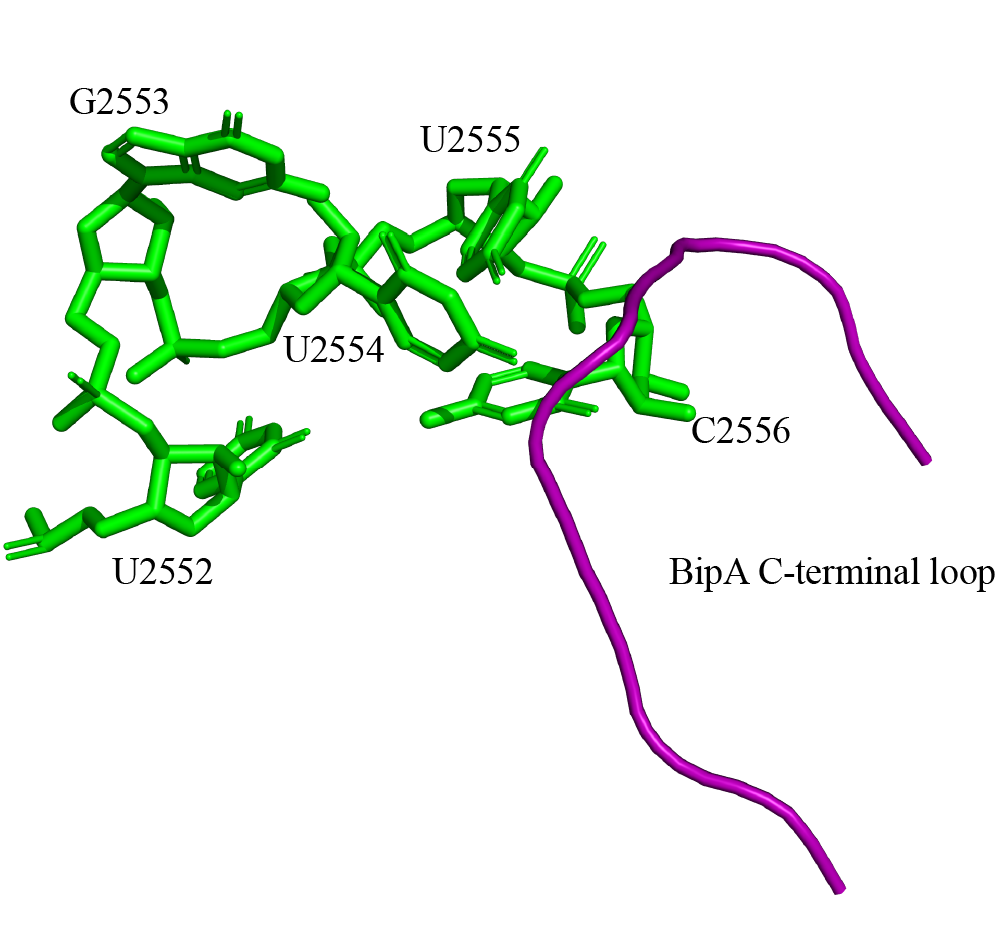

Supplement: Supplementary file 5 [file Data_Sheet_1.docx]
